# *Vibrio cholerae* Response Regulator VxrB Controls Colonization and Regulates the Type VI Secretion System

**DOI:** 10.1371/journal.ppat.1004933

**Published:** 2015-05-22

**Authors:** Andrew T. Cheng, Karen M. Ottemann, Fitnat H. Yildiz

**Affiliations:** Department of Microbiology and Environmental Toxicology, University of California, Santa Cruz, California, United States of America; University of California, Davis, UNITED STATES

## Abstract

Two-component signal transduction systems (TCS) are used by bacteria to sense and respond to their environment. TCS are typically composed of a sensor histidine kinase (HK) and a response regulator (RR). The *Vibrio cholerae* genome encodes 52 RR, but the role of these RRs in *V*. *cholerae* pathogenesis is largely unknown. To identify RRs that control *V*. *cholerae* colonization, in-frame deletions of each RR were generated and the resulting mutants analyzed using an infant mouse intestine colonization assay. We found that 12 of the 52 RR were involved in intestinal colonization. Mutants lacking one previously uncharacterized RR, VCA0566 (renamed VxrB), displayed a significant colonization defect. Further experiments showed that VxrB phosphorylation state on the predicted conserved aspartate contributes to intestine colonization. The VxrB regulon was determined using whole genome expression analysis. It consists of several genes, including those genes that create the type VI secretion system (T6SS). We determined that VxrB is required for T6SS expression using several in vitro assays and bacterial killing assays, and furthermore that the T6SS is required for intestinal colonization. *vxrB* is encoded in a four gene operon and the other *vxr* operon members also modulate intestinal colonization. Lastly, though Δ*vxrB* exhibited a defect in single-strain intestinal colonization, the Δ*vxrB* strain did not show any *in vitro* growth defect. Overall, our work revealed that a small set of RRs is required for intestinal colonization and one of these regulators, VxrB affects colonization at least in part through its regulation of T6SS genes.

## Author Summary

Pathogenic bacteria experience varying conditions during infection of human hosts and often use two-component signal transduction systems (TCSs) to monitor their environment. TCS consists of a histidine kinase (HK), which senses environmental signals, and a corresponding response regulator (RR), which mediates a cellular response. The genome of the human pathogen *V*. *cholerae* contains a multitude of genes encoding HKs and RRs proteins. In the present study, we systematically analyzed the role of each *V*. *cholerae* RR for its role in pathogenesis. We identified a previously uncharacterized RR, VxrB, as a new virulence factor. We demonstrated that VxrB controls expression of the type VI secretion system (T6SS), a virulence nanomachine that directly translocates effectors into bacterial or host cells, thereby facilitating colonization by competing with sister cells and intestinal microbiota. This study represents the first systematic analysis of the role of all RRs in *V*. *cholerae* pathogenesis and provides a foundation for understanding the signal transduction pathways controlling *V*. *cholerae* pathogenesis.

## Introduction

*Vibrio cholerae* causes the diarrheal disease cholera that affects 3 to 5 million people worldwide every year, resulting in 100,000–120,000 deaths annually [1]. *V*. *cholerae* produces a number of virulence factors which facilitate colonization of the intestine and subsequent disease. Major virulence factors are cholera toxin (CT), which is responsible for production of profuse watery diarrhea, and a type IV pilus called the toxin-coregulated pilus (TCP), which is required for intestinal colonization [2]. *V*. *cholerae* virulence factors are well known to be under extensive transcriptional control. CT and TCP production are controlled by the transcriptional activator ToxT [3, 4]. Expression of *toxT*, in turn, is controlled by a virulence regulatory cascade involving the membrane-bound transcriptional activators ToxRS and TcpPH. These two regulators activate *toxT* transcription directly [5–7]. TcpPH expression is activated by the transcriptional activators AphA and AphB [8, 9]. The quorum sensing (QS) regulatory system is also linked to the virulence gene regulatory cascade through HapR, the master QS regulator, which represses *aphA* expression [10].

Recently, the type VI secretion system (T6SS) has been identified as a new virulence factor in *V*. *cholerae* [11, 12]. T6SSs deliver effector proteins into both eukaryotic and bacterial cells in a contact-dependent manner [12, 13]. *V*. *cholerae* has one T6SS system with multiple T6SS effectors: VrgG1 and VrgG3 (valine-glycine repeat protein G), which have actin cross-linking activity and peptidoglycan-degrading activity, respectively [14–17]; TseL, which has lipase activity [15, 18]; and VasX, which perturbs the cytoplasmic membrane of target cells [15, 19]. Activity of these effectors is antagonized by corresponding immunity proteins: TsiV3, TsiV1, and TsiV2, respectively, to prevent killing by strains bearing these proteins [15, 16, 20, 21].

The T6SS can be divided into functional sections consisting of the core structural components, the T6SS effector and immunity proteins, and transcriptional regulators. The base of the T6SS apparatus spans the cell envelope, and is a tube within a tube. The inner tube is composed of polymers of the hemolysin coregulated protein (Hcp). The outer tube, also called the contractile sheath, is formed by polymers of VipA and VipB [14, 22]. The Hcp inner tube is capped with a spike complex of trimeric VgrG proteins. The effectors are delivered by contraction of the VipA/VipB sheath, which in turn results in ejection of the inner tube along with VgrG and the effectors towards the target cell [12].

The genes encoding the T6SS components are organized into one large cluster (VCA0105-VCA0124) and two auxiliary clusters (VCA0017-VCA0022 and VC1415-VC1421) [11, 23]. A key positive transcriptional regulator of the *V*. *cholerae* T6SS is VasH (VCA0117), which is related to enhancer binding proteins that activate transcription in a σ54 (RpoN) dependent manner [24, 25]. VasH acts on the T6SS auxiliary clusters and *vgrG3* of the large cluster, but does not affect expression of the structural genes encoded in the large T6SS gene cluster [24, 26]. Additionally, Hcp production is positively regulated by the master quorum sensing regulator HapR and the global regulator cyclic AMP (cAMP) receptor protein CRP, and negatively regulated by QS regulator LuxO and by global regulator TsrA, a protein homologous to heat-stable nucleoid-structuring (H-NS) [27, 28]. These studies have thus shown that numerous global regulators control T6SS expression, as well as one specific regulator (VasH).

*V*. *cholerae* T6SS studies have mainly focused on the *V*. *cholerae* O37 serogroup V52 strain because it assembles a T6SS apparatus constitutively [11]. In this strain, the T6SS is required for cytotoxicity towards *Dictyostelium discoideum* and J774 macrophages, and induces inflammatory diarrhea in the mouse model [29]. In *V*. *cholerae* O1 strain C6706, the T6SS is not constitutively produced and conditions that promote T6SS production are unknown. However, production of T6SS can be achieved in other O1 strains by inactivating mutations in genes encoding the LuxO and TsrA negative regulators. In O1 strains, the T6SS translocates T6SS effectors into macrophages, and increases fecal diarrhea and intestinal inflammation in infant rabbits [27]. It was also shown that the *V*. *cholerae* O1 C6706 strain T6SS mediates antagonistic interbacterial interactions during intestinal colonization. A strain unable to produce the TsiV3 immunity protein, which provides immunity against the effector VgrG3, exhibited an intestinal colonization defect only when co-infected with strains harboring an intact T6SS locus and VrgG3 [30]. Although T6SS is regulated and expressed differently between *V*. *cholerae* strains, production of this system in multiple strains promotes virulence against both eukaryotic and bacterial cells, suggesting the function is largely conserved but the regulation varies.

Pathogenic bacteria experience varying conditions during infection of human hosts and often use two-component signal transduction systems (TCSs) to monitor their environments [31]. TCSs play important roles in the regulation of virulence factors, metabolic adaptation to host environments, and response to numerous environmental stresses including pH, osmolarity, oxygen availability, bile salts, and antimicrobial peptides [32]. TCS rely on a phosphorelay-based signal transduction system. The prototypical TCS consists of a membrane-bound histidine kinase (HK), which senses environmental signals, and a corresponding response regulator (RR), which mediates a cellular response. Response regulators are typically multi-domain proteins harboring a conserved receiver domain (REC) and C-terminal output domain such as DNA-binding, diguanylate cyclase, or methyltransferase [33–35]. Upon environmental stimulation, the HK catalyzes an ATP-dependent autophosphorylation reaction on a conserved histidine residue. The phosphoryl group is transferred from the HK to a conserved aspartate residue on the RR, eliciting a conformation change and subsequent cellular response [32, 34, 35].

The *V*. *cholerae* genome reference genome of O1 EL Tor N16961 strain is predicted to encode 43 HK and 49 RR (http://www.ncbi.nlm.nih.gov/Complete_Genomes/RRcensus.html and http://www.p2cs.org). We also included 3 additional RRs (VpsT, VpsR, QstR) which were not annotated in these databases. Thirteen of these 52 putative RRs have been previously characterized and eight have a role in virulence factor production and host colonization: VarA, LuxO, VieA, PhoB, ArcA, FlrC, CarR, and CheY-3 [36–44]. VarA and LuxO repress production of quorum sensing regulator HapR, which represses expression of *aphA* and, in turn, TCP and CT production [36, 37], VieA regulates *ctxAB* expression indirectly by affecting production of ToxT through cyclic diguanylate (c-di-GMP) signaling [38, 39]. The RR for phosphate limitation, PhoB, directly controls expression of a key transcriptional regulator, TcpPH, which activates *toxT* transcription [40]. The RR ArcA controls adaptation to low oxygen environment of the intestine and positively controls the expression of *toxT* [41]. CarR regulates glycine and diglycine modification of lipid A, confers polymyxin B resistance, and is required for intestinal colonization, although this phenotype is strain dependent [42]. FlrC controls flagellar biosynthesis and CheY-3 is needed for control of chemotactic motility [43, 44]. Both motility and chemotaxis are known colonization factors for *V*. *cholerae* [43]. Together, these results show that RRs shown play a role in intestinal colonization have three basic targets: known virulence regulators and concomitant CT and TCP production; lipid A modification enzymes; or motility and chemotaxis. 39/52, however, were not yet analyzed at the time of this study.

To systematically evaluate the role of *V*. *cholerae* TCSs in intestinal colonization, we generated in-frame deletion mutants of each RR gene and analyzed the *in vivo* colonization phenotypes of the resulting mutants. We found 12 RR were required for wild-type intestinal colonization. One RR in particular had a very strong defect, encoded by genomic locus VCA0566. We determined that VCA0566 (now termed *V* *ibrio* type six secretion regulator, *vxrB*) controls expression of several genes including the T6SS genes. We used multiple methods to substantiate that VxrB is required for expression of the T6SS *in vitro* and *in vivo*. Lastly, we report that the T6SS contributes to colonization of the *V*. *cholerae* O1 strain used in this study.

## Results

### Multiple RRs impact intestinal colonization

**Fig 1:**
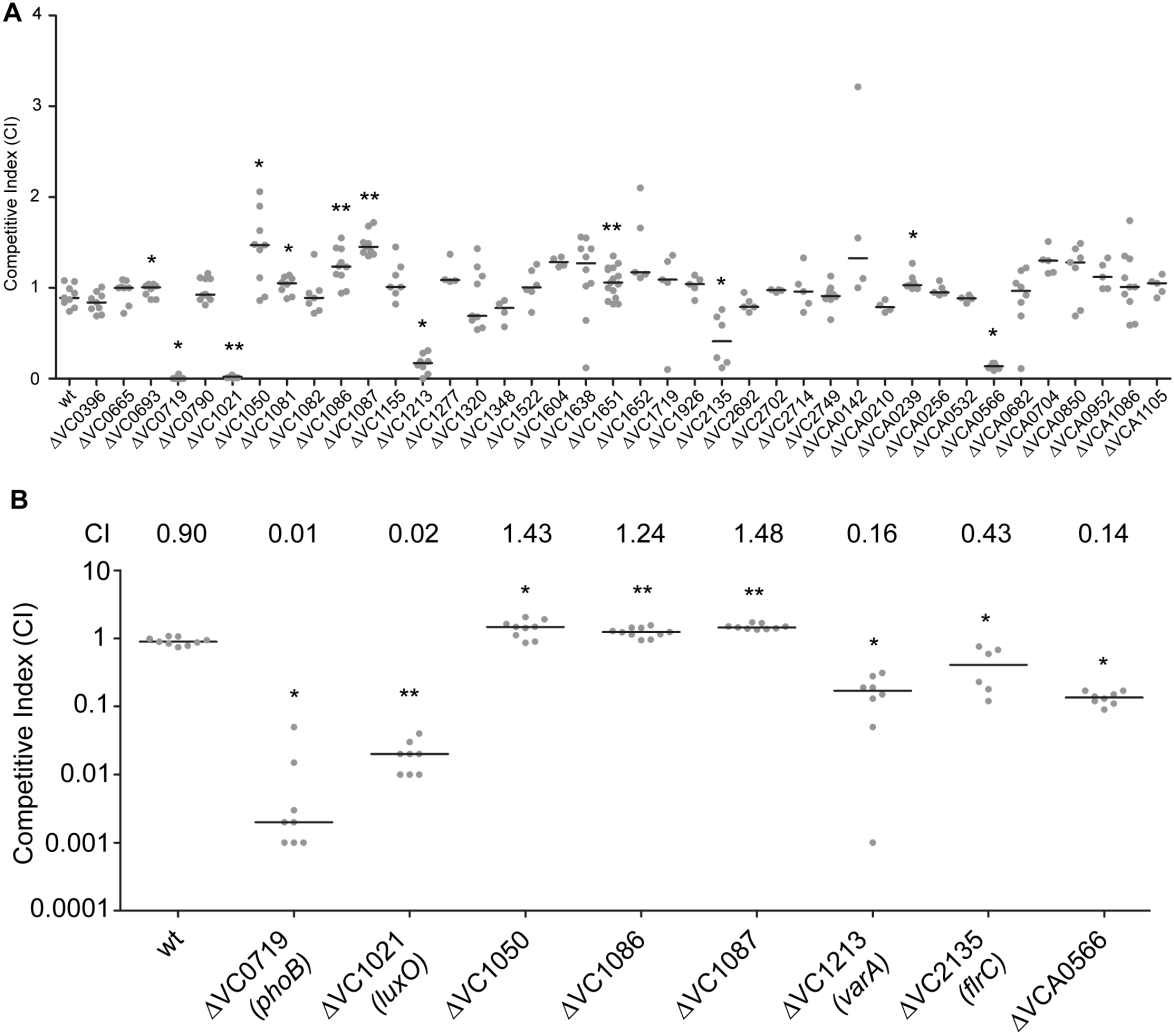
Identification of RRs impacting colonization in the infant mouse infection model. (A) Ability of 40 ΔRR mutants in *V*. *cholerae* strain A1552 to colonize the infant mouse intestine was analyzed using a competition assay with the isogenic wild-type strain. (B) Same data presented in Fig 1A, expanded to highlight the mutants showing a statistically significant difference in colonization p<0.05 and a minimum 1.2-fold change in colonization ability. Competitive index (CI) is defined as the output ratio of mutant to wild-type bacteria divided by the input ratio of mutant to wild-type bacteria. Each symbol represents the CI in an individual mouse; horizontal bars indicate the median. Statistical analysis was carried out using Wilcoxon Signed Rank Test, comparing the CI of each strain to the CI of wt *lacZ* ^+^ / wt lacZ^-^ (shown as wt) (*, p<0.05; **, p<0.01).

We have a limited understanding of the *V*. *cholerae* TCSs and their role in colonization and adaptation to host environments. To evaluate the importance of the 52 TCS RRs in colonization, we generated in-frame deletion mutants of the 40 RRs. For this analysis, we excluded 12 RR that were either predicted to be involved in chemotaxis (11 CheY, CheV, and CheB proteins) or that we were unable to mutate (VC2368, ArcA) [43, 45]. We then analyzed the ability of 40 RR deletion mutants to colonize the small intestine in an *in vivo* competition assay where *in vivo* fitness of a mutant strain is compared to that of wild type strain using the infant mouse infection model (Fig 1A) [46]. While the vast majority of mutants—28—were not different from wild type, we identified 12 RR mutants that had a statistically significant colonization difference as compared to wild type (Fig 1A). We focused on 8 mutants with a statistically significant colonization difference and exhibited at least 1.2-fold difference in CI (Fig 1B). Consistent with previous studies, we identified that ΔVC0719 (*phoB)*, ΔVC1021 *(luxO)*, ΔVC1213 (*varA)*, *and* ΔVC2135 *(flrC)* were defective in colonization [36, 37, 40, 44]. The competitive indices (CI) for Δ*phoB*, Δ*luxO*, Δ*varA*, and Δ*flrC* were 0.01, 0.02, 0.16, and 0.43, respectively (Fig 1B).

Additionally, we identified a set of genes whose absence slightly but statistically significantly enhanced colonization (at least 1.2 fold higher CI), suggesting that inhibition of their expression and activity may be needed for wild-type colonization. These mutants were ΔVC1050, ΔVC1086, and ΔVC1087, which exhibited subtle and enhanced colonization phenotypes with CIs of 1.43, 1.24, and 1.48, respectively (Fig 1B). VC1050 is classified as an Hnr-type RR, [47] but its function is yet to be determined. VC1086 and VC1087 are part of a predicted eight gene operon encompassing VC1080-VC1087. Both VC1086 and VC1087 have domains that suggest they function in cyclic guanylate (c-di-GMP) regulation. Specifically, VC1086 contains an EAL domain with conserved residues required for enzymatic function, while VC1087 harbors an HD-GYP domain, but this domain lacks the conserved residues required for enzymatic activity.

We also identified one RR that was defective for colonization that had not been previously characterized. This mutant, ΔVCA0566, had a colonization defect with a CI of 0.14 (Fig 1A and 1B). Because this uncharacterized RR was important for colonization, we focused the rest of our studies on this protein.

### ΔVCA0566/VxrB impacts colonization

**Fig 2:**
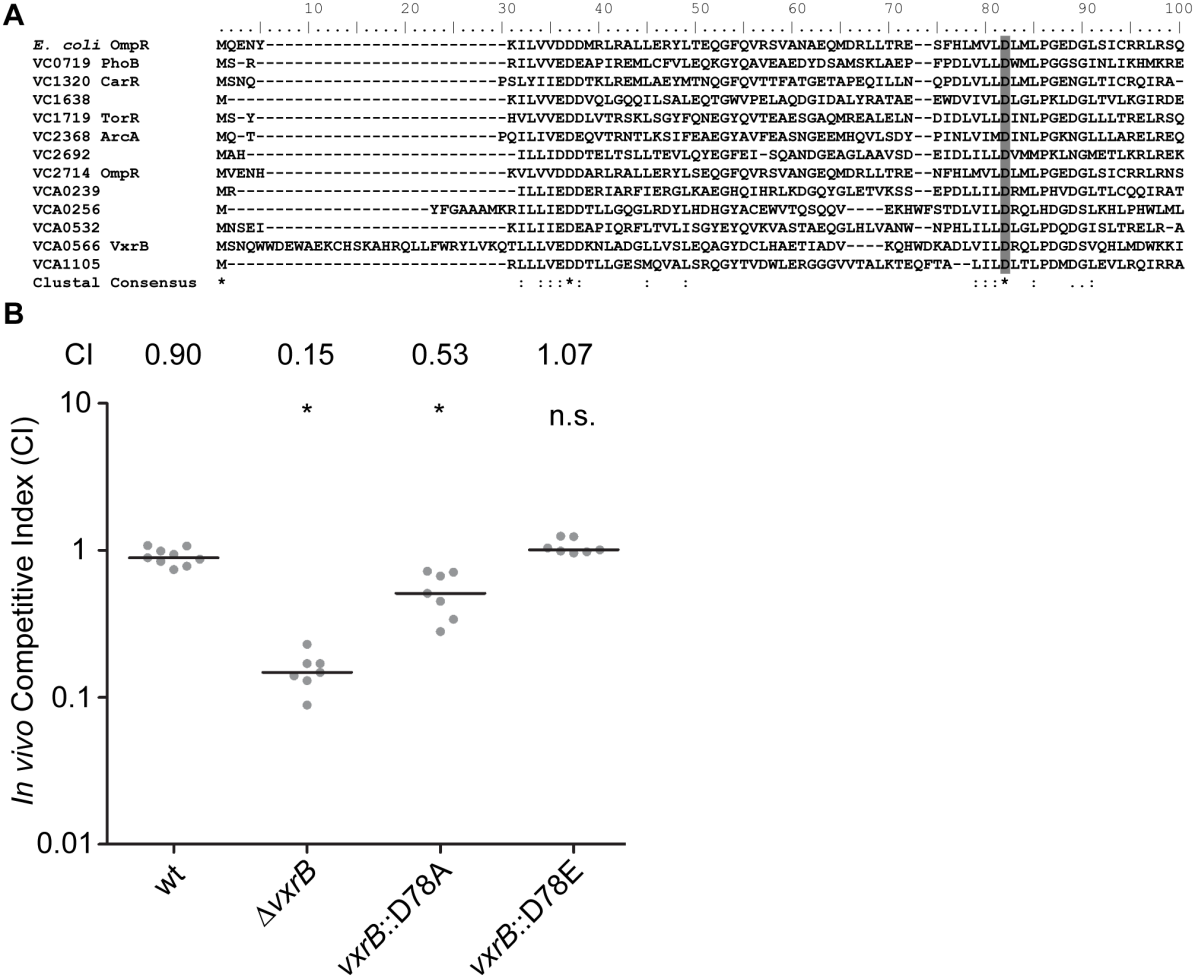
The role of the phosphorylation state of VxrB in colonization. (A) Amino acid sequence alignment of the REC domains of the RR proteins belonging to the OmpR family using ClustalW. Numbers above the sequence correspond to the amino acid number of each protein. VC numbers indicate the RR encoded in *V*. *cholerae* genome. OmpR from *E*. *coli* was used as a reference to align the aspartate residue that has been shown to be important for phosphorylation (D55A) [48]. Highlighted region in gray indicate the aspartate residue that is predicted to be the site of phosphorylation. B) *In vivo* competition assay of a strain harboring the mutated version of *vxrB* on the chromosome. The *vxrB* gene was mutated to convert the aspartate residue predicted to be important for phosphorylation to emulate the inactive (D78A) or active (D78E) state of VxrB. *, p<0.05 by the Wilcoxon Signed Rank Test as compared to the CI of wt.

VCA0566 is the second gene of a predicted five gene operon and had been previously annotated as a RR of the OmpR family. The encoded protein, which we named VxrB for reasons described below, is 245 amino acids in length with an N-terminal REC domain and a C-terminal winged helix-turn-helix DNA-binding domain (Fig 2B). Previously characterized members of the OmpR family in *V*. *cholerae* include PhoB, CarR, and ArcA [40–42]. Amino acid sequence alignment of the *V*. *cholerae* RRs in the OmpR family and the previously characterized *E*. *coli* OmpR [48] was used to identify the aspartate residue that is predicted to be phosphorylated in the REC domain (Fig 2A). Since the phosphorylation state of a RR is likely to determine its activity, we mutated the aspartate residue in the REC domain of VxrB to mimic constitutively active (D78E) and inactive (D78A) versions, as used in other work [48], and replaced the wild-type gene in the chromosome with these altered genes. These mutants were competed against wild type in the infant mouse colonization assay to determine if the phosphorylation state of VxrB is important for colonization. In accordance with our initial colonization screen, Δ*vxrB* displayed a CI of 0.15 (Fig 2B). Somewhat surprisingly, the CI for *vxrB*::D78A (inactive form) was 0.53, indicating a modest defect in colonization. This result indicates that the “inactive” form of VxrB does not phenocopy the Δ*vxrB* mutant, suggesting that VxrB harboring D78A substitution is not fully inactive. The CI for *vxrB*::D78E (active form) is 1.07, suggesting that constitutive activation of VxrB does not significantly impair *V*. *cholerae* (Fig 2B). Collectively, these findings suggest that in vivo phosphorylation of VxrB at D78 is likely to be important for its colonization function, but apparently not absolutely required. It is also likely that VxrB may not function by conventional phosphorylation-dependent signal transduction [49].

### All members of the *vxr* operon contribute to *in vivo* colonization

**Fig 3:**
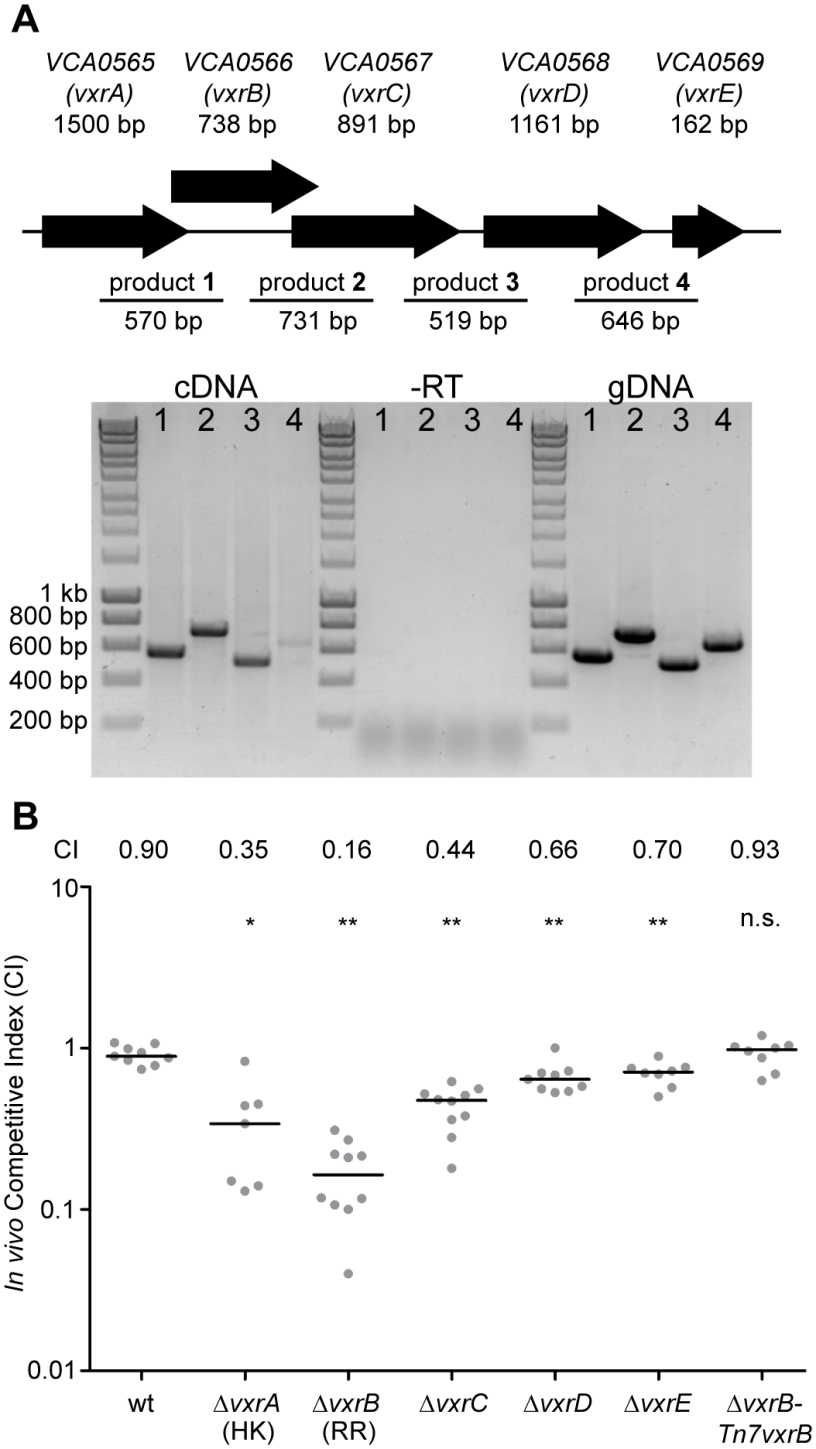
Role of the *vxrABCDE* operon in colonization. (A) The top panels shows a schematic representation of the genomic organization of the *vxrABCDE* locus The bottom panel shows the results of RT-PCR analysis designed to determine if *vxrABCDE* genes are cotranscribed. RT-PCR primers designed to amplify intergenic regions are indicated. Products were detected for VCA0565-66 (product 1), VCA0566-67 (product 2), VCA0667-68 (product 3), and VCA0568-69 (product 4) *V*. *cholerae* genomic DNA template was used for PCR to evaluate primers and amplified product sizes. RT-PCR reaction without reverse transcriptase (-RT) was used as a negative control. (B) Ability of A1552 Δ*vxrA*, Δ*vxrB*, Δ*vxrC*, Δ*vxrD*, Δ*vxrE*, and Δ*vxrB*-Tn7*vxrB* complementation strains to colonize the infant mouse intestine was analyzed using a competition assay with wild-type. Each symbol represents the CI in an individual mouse; horizontal bars indicate the median. *, p<0.05; **, p<0.01 by the Wilcoxon Signed Rank Test as compared to the CI of wt.

The first gene of the *vxr* loci, VCA0565, is annotated as an HK. The other three genes (VCA0567-69) are predicted to encode proteins of unknown function (Fig 3A). We now termed these genes *V* *ibrio* type six secretion regulator (*vxr*) ABCDE and determined that these genes are co-transcribed using RT-PCR and RNAseq analysis (Fig 3A and S1 Fig). Both the genomic context and organization is conserved in the *Vibrio* species (S2–S4 Figs) and *vxr* gene products do not share significant sequence similarity with previously characterized proteins.

To gain a better understanding of the role of the *vxr* operon in colonization, we investigated whether the cognate HK and other genes in the *vxr* operon also contributed to mouse colonization. In-frame unmarked deletion mutants of *vxrA*, *vxrB*, *vxrC*, *vxrD*, and *vxrE* (Fig 3B) were generated and analyzed in an *in vivo* competition assay. Each mutant was outcompeted, with CIs of 0.35, 0.16, 0.44, 0.66, and 0.70, respectively (Fig 3B). These findings suggest that while *vxrA and vxrB* genes are critical for colonization in the infant mouse model, contribution of *vxrCDE* genes appears to be minor.

To further confirm the phenotype of Δ*vxrB* colonization defect, a wild type copy of *vxrB* whose expression was driven from its native promoter was inserted into the Tn7 site (located between VC0487 and VC0488) on the chromosome of Δ*vxrB*. *In vivo* competition assay of Δ*vxrB-Tn7vxrB* had a CI of 0.93, similar to wild type levels, where Δ*vxrB* had a CI of 0.16 (Fig 3B). Thus, the Δ*vxrB* colonization defect is restored to wild-type levels by introduction of the wild-type copy of *vxrB*.

### VxrB regulates T6SS gene expression

To gain a better understanding of the contribution of VxrB to *V*. *cholerae* pathogenesis, we performed high throughput transcriptome sequencing (RNA-seq) analysis to identify the *V*. *cholerae* genes controlled by VxrB. We used cells grown under virulence inducing AKI conditions, to mimic the intestinal conditions encountered when we know VxrB is important. 149 genes showed statistically significant differences in gene expression between the wild type and mutant (S2 and S3 Tables). Of these, 80 genes were expressed to greater levels in the Δ*vxrB* mutant relative to the wild type (S2 Table), while 69 were expressed to lower levels in the Δ*vxrB* mutant relative to wild type (S3 Table). Of particular interest was the observation that message abundance of most of the T6SS genes in both the large cluster (VCA0105-VCA0124) and the two auxiliary clusters (VCA0017-VCA0022 and VC1415-VC1421) were less in the VxrB mutant relative to wild type (Table 1) (S5 Fig). This finding suggests that VxrB activates expression of the T6SS genes.

**Table 1:** Expression of Type VI secretion genes in the Δ*vxrB* mutant relative to wild type.

ORF ID	Gene	AKI	
		Fold change	P-value
VCA0105		-4.44	0.00000
VCA0106		-2.97	0.00000
VCA0107	*vipA*	-2.11	0.00113
VCA0108	*vipB*	-2.05	0.00000
VCA0109		-2.39	0.00007
VCA0110	*vasA*	-1.59	0.00002
VCA0111	*vasB*	-1.99	0.00001
VCA0112	*fha*	-2.72	0.00000
VCA0113	*vasD*	-2.29	0.00000
VCA0114	*vasE*	-1.89	0.00000
VCA0115	*vasF*	-1.85	0.00001
VCA0116	*clpB-2*	-2.05	0.00000
VCA0117	*vasH*	-1.87	0.00000
VCA0018	*vasI*	-1.46	0.00612
VCA0119	*vasJ*	-1.77	0.00000
VCA0120	*vasK*	-1.95	0.00000
VCA0121	*vasL*	-1.96	0.00000
VCA0122	*vasM*	-2.17	0.02985
VCA0123	*vgrG-3*	-1.50	0.00000
VCA0124	*tsiV3*	-1.72	0.00144
VCA0017	*hcp2*	-2.82	0.00000
VCA0018	*vgrG-2*	-1.22	0.41237
VCA0019	*vasW*	1.15	0.67767
VCA0020	*vasX*	-1.33	0.11525
VCA0021	*tsiV2*	-1.38	0.11525
VC1415	*hcp1*	-3.02	0.00000
VC1416	*vgrG-1*	-1.12	0.50510
VC1417		-1.44	0.04444
VC1418	*tseL*	-1.29	0.06289

### VxrB regulates production of T6SS

**Fig 4:**
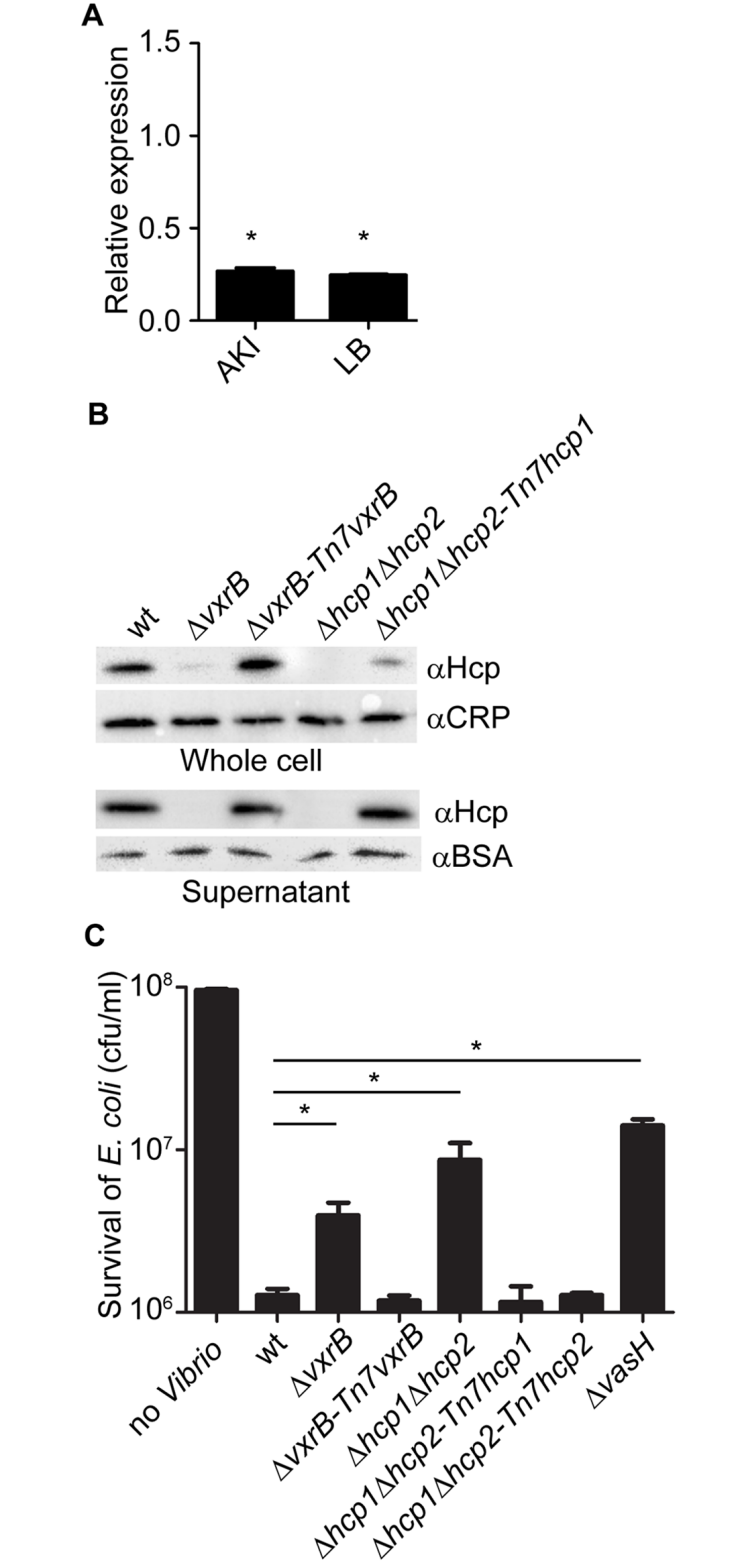
Analysis of Hcp production and secretion in the *vxrB* mutant. (A) Quantitative real-time PCR analysis of *hcp* using total RNA isolated from wild-type and Δ*vxrB* grown in AKI and LB. Experiments were performed using two independent biological replicates, each performed in quadruplicate. The Pfaffl method was used to compare expression levels of *hcp* to 16s rRNA and relative expression was calculated by comparing expression in Δ*vxrB* with that of wt. *, p<0.05 by Student’s t-test. (B) Hcp production and secretion was analyzed in whole cells and culture supernatants in wild type, Δ*vxrB*, Δ*vxrB*::*Tn7vxrB*, Δ*hcp1*Δ*hcp2*, and Δ*hcp1*Δ*hcp2*::*Tn7hcp1* strains by immunoblot analysis. Equal amounts of total protein (determined by BCA assay) were loaded onto a SDS 13% polyacrylamide gel. Prior to TCA precipitation and total protein quantification, 100μg/ml of BSA was added to the supernatant. After the blot was probed for Hcp, the blot was stripped of all antibodies using Western Blot Stripping Buffer (GM Biosciences, MD) and the same blot was used to probe for CRP and BSA in the whole cell and supernatant, respectively. The data shown is representative of the results of three independent experiments. (C) Interbacterial killing was analyzed by mixing *V*. *cholerae* strains and prey *E*. *coli* strain MC4100 in a 10:1 ratio, followed by incubation on LB agar plates for 4 hours at 30°C and determination of surviving *E*. *coli* MC4100. The data represent averages and standard deviations of three independent experiments. *, p<0.05 by the Student’s t-test as compared to the values for interbacterial killing of the wild-type strain.

To further analyze the role of *vxrB* in T6SS expression and function, we compared the levels of the major T6SS structural component, Hcp, between wild type and Δ*vxrB* mutant *V*. *cholerae*. Quantitative real-time PCR analysis of *hcp* revealed that the transcript abundance of *hcp* was decreased by 3.7-fold under AKI conditions and 4.1- fold under LB conditions in the Δ*vxrB* mutant relative to wild type (Fig 4A). This finding supports that VxrB regulates expression of *hcp* and is consistent with the RNA-seq analysis. Additionally the levels of the Hcp protein in Δ*vxrB* were lower than wild type, in both whole cell samples and culture supernatants (Fig 4B). We also determined that complementation of the *vxrB* mutation (Δ*vxrB-Tn7vxrB*) restored Hcp to wild-type levels. Because we found lower amounts of Hcp in the supernatant as well as in whole cells, this finding suggests that VxrB is needed to express and secrete Hcp. As negative controls, we included a Δ*hcp1*Δ*hcp2* mutant because it is unable to produce the Hcp proteins [11, 50]. As expected, no Hcp production was observed in this mutant. Furthermore, complementation of *hcp1* in the Δ*hcp1*Δ*hcp2* mutant partially restored Hcp levels (Fig 4B). Overall these findings suggest that Hcp production is decreased in Δ*vxrB* mutant.

Next we analyzed whether VxrB was needed for T6SS function, by examining T6SS-mediated interbacterial killing. Killing assays between the *V*. *cholerae* and the target *E*. *coli* K-12 strain MC4100 showed that wild-type *V*. *cholerae* decreased the numbers of *E*. *coli* compared to control experiments. This killing was dependent on the T6SS, as shown by greater numbers of *E*. *coli* obtained when incubated with *V*. *cholerae* Δ*hcp1*Δ*hcp2* mutant and Δ*vasH* mutants, consistent with the findings reported by Ishikawa *et al*. (Fig 4C) [50]. This phenotype was complemented by introduction of either *hcp1* or *hcp2* into the Tn7 site on the chromosome. Consistent with our transcriptional and protein analysis presented above, we found that Δ*vxrB* mutants mediated less *E*. *coli* killing. These findings suggest that T6SS regulation by VxrB contributes to interbacterial killing.

**Fig 5:**
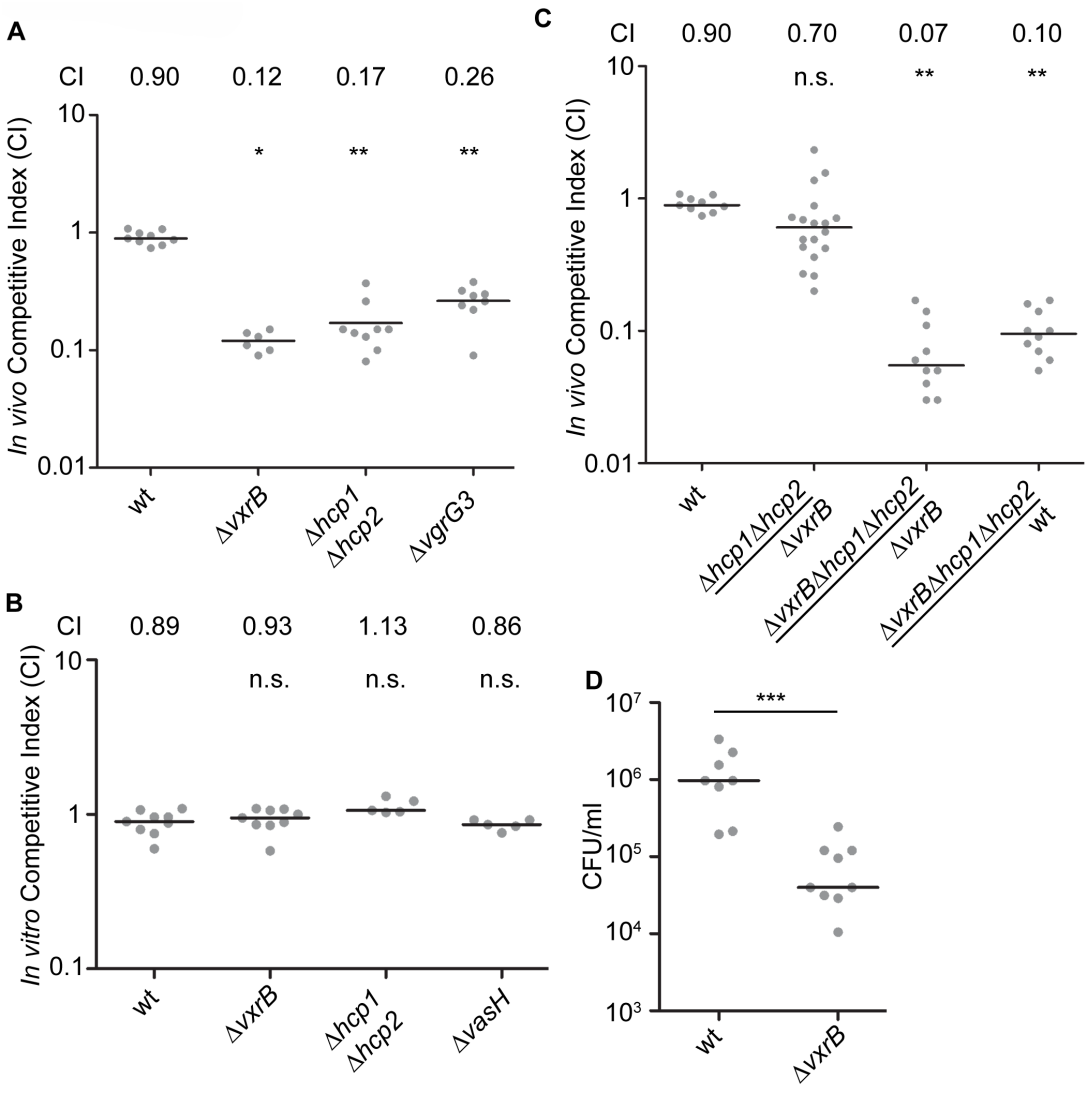
Analysis of the role of T6SS in colonization. (A) Ability of Δ*vxrB*, Δ*vxrB*-Tn7*vxrB*, Δ*hcp1*Δ*hcp2*, Δ*vgrG3*, to colonize the infant mouse intestine was analyzed using a competition assay with isogenic wild-type strain. *, p<0.05; **, p<0.01 by the Wilcoxon Signed Rank Test as compared to wt (CI of 0.90). (B) *In vitro* competition assay of *ΔvxrB*, Δ*hcp1*Δ*hcp2*, and *ΔvasH*. Strains were grown in LB at 30°C for 20 hours. Statistical analysis was carried out using Wilcoxon Signed Rank Test, comparing the CI of each strain to the CI of wt *lacZ* ^+^ / wt lacZ^-^ (shown as wt) (CI of 0.98). (C) Ability of Δ*hcp1*Δ*hcp2* to colonize the infant mouse intestine was analyzed using a competition assay with Δ*vxrB*. (D) Ability of wild type and Δ*vxrB* to colonize the infant mouse intestine in single-strain infections. Each symbol represents the CI in an individual mouse; horizontal bars indicate the median. ***, p<0.001 by the Student’s t-test as compared to the values for colonization of the wild-type strain. n.s. indicates mutants were not significantly different from the wt.

Since VxrB regulates T6SS expression and is required to for intestinal colonization, we next asked whether the T6SS itself is required for intestinal colonization. We performed *in vivo* competition assays of a T6SS null mutant (Δ*hcp1*Δ*hcp2*) against wild type in the infant mouse model. We found that the *in-vivo* CI for Δ*hcp1*Δ*hcp2* was 0.17 (Fig 5A). In addition, Δ*vgrG3* also had an *in-vivo* CI of 0.26 suggesting that T6SS components are important for intestinal colonization (Fig 5A). This suggests that structural components of the T6SS are needed to colonize the intestine. Furthermore, this finding also suggests that the colonization defect associated with the Δ*vxrB* mutant could be caused by diminished T6SS production. To evaluate this possibility, we tested the *in vivo* competition of Δ*hcp1*Δ*hcp2* against Δ*vxrB* and found that these strains competed nearly equally with each other (Fig 5C). Furthermore, in-vivo CI of Δ*vxrB*Δ*hcp1*Δ*hcp2* triple mutant against Δ*vxrB* was 0.07 and Δ*vxrB*Δ*hcp1*Δ*hcp2* against wt was 0.10. This finding suggests that the colonization defect by Δ*vxrB* was not solely due to altered expression of T6SS genes and other factors regulated by VxrB also contribute to colonization. It is also likely that T6SS expression is not completely abolished by the *vxrB* mutation. Indeed, western analysis (Fig 4B) shows that in *vxrB* mutant Hcp production is reduced but not completely eliminated. Similarly in vitro killing assay shows that *vxrB* mutant’s interbacterial killing ability is not identical to that of the strain lacking T6SS.

We next asked whether VxrB plays a role in growth in vitro, by performing an *in vitro* competition assay. Δ*vxrB* mutants grew equally well as wild type, suggesting that neither had a competitive advantage over the other *in vitro* (Fig 5B). This outcome suggests that there may be an *in vivo* signal produced in the infant mouse that triggers T6SS activity and colonization. We also performed single-strain colonization assays in the infant mouse model with *ΔvxrB*. There was a 12.7-fold decrease in colonization for Δ*vxrB* compared to wild type (Fig 5D). This finding suggests that the colonization defect by Δ*vxrB* was not solely dependent on wild type, and possibly could be caused by competition with the normal flora or ability of the mutant to adapt to the infection microenvironment.

## Discussion

Systematic mutational phenotypic characterization of TCSs has been performed in only a few bacteria, including *Vibrio fischeri*, *E*. *coli*, *Bacillus subtilis*, *Streptococcus pneumoniae*, and *Enterococcus faecalis* [51–55]. In this study, we systematically analyzed the role of all *V*. *cholerae* TCS in colonization of the infant mouse small intestine and identified the RRs that play roles in mouse intestinal colonization. Specifically, ΔVC0719 (*phoB*), ΔVC1021 (*luxO*), ΔVC1213 (*varA*), *and* ΔVC2135 (*flrC*), and ΔVCA0566 (*vxrB*) exhibited intestinal colonization defects while ΔVC1050, ΔVC1086, and ΔVC1087 showed enhanced colonization. Many of the RRs had either no statistically significant defect or minor defects in the infant mouse colonization assay. It remains possible, however, that these RRs have a role in colonization in other infection models.

*In vivo* transcriptome analysis has been performed on different strains of *V*. *cholerae* in the infant mouse and rabbit ileal loop infection models. The analysis of the whole genome expression of *V*. *cholerae* O1 El Tor C6706 cells accumulating in the ceca of orally infected infant rabbits and the intestines of orally infected infant mice revealed that expression of the genes encoding RRs is altered during *in vivo* growth conditions as compared to *in vitro* growth in nutrient broth and that *in vivo* expression of TCS also differed between the model systems [56]. In the infant rabbit infection model, expression of seven RR (VC1081, VC1082, VC1155, *vieA*, VC2702 (*cbrR*), VCA0210, and VCA1105) was increased and 1 RR (*carR*) was decreased by more than 2-fold significantly in comparison to *V*. *cholerae* cells grown *in vitro* in nutrient broth. In the infant mouse infection model, expression of 17 RR (*vpsR*, VC1050, VC1081, VC1082, VC1086, VC1087, VC1155, VC1522, *flrC*, *cbrR*, *ompR*, *dct-D2*, *vxrB*, *uhpA*, *vpsT*, VCA1086, and VCA1105) and 9 RR (*qstR*, *phoB*, VC1348, VC1638, *vieB*, *cpxR*, *ntrC*, VCA0532, *pgtA*) were either decreased and increased significantly by more than 2-fold, respectively, in comparison to *V*. *cholerae* cells grown *in vitro* in nutrient broth [56]. *vxrA* and *vxrB* transcript levels were decreased 2 and 3-fold, respectively, in the experiments reported by Mandlik and colleagues, but did not reach statistical significance [56]. This work all used the *V*. *cholerae* O1 El Tor strain C6706, and so it is yet unknown whether *vxrB* expression is similarly regulated in the O1 El Tor A1552 strain used here.

There have been two other studies that analyzed *V*. *cholerae* infection phenotypes on a global scale, although they did not specifically target RR. Together, these studies and ours suggests there is a set of genes required for intestinal colonization across multiple models. Fu *et al*. used random transposon mutants coupled with insertion site sequencing (Tn-seq) in a rabbit model [30]. They identified insertions in two genes—VC1021 (*luxO*) and VC1155—that showed 8-15-fold reduction in colonization, while strains harboring insertions into RRs VC1348, *vieA*, *vieB*, *arcA*, VCA0256, *uhpA*, and *pgtA* had less than a 5-fold reduction in colonization (p<0.001). Another Tn-seq study using the infant rabbit model identified defects associated with *luxO* and *arcA* as above, and additionally *phoB* and *varA* [57]. Combining the results of these studies with ours identifies *luxO*, *phoB*, and *varA*, as required for *in vivo* fitness, and others that are variably identified. Because the Tn-seq work used transposon libraries, it is not known whether all RR were eliminated, so it is possible that their studies missed some critical RR. There are hints in their data, however, that the *vxr* locus is necessary in these other models as well. While Fu *et al*. did not identify *vxrA* or *vxrB* mutants, they did determine that a strain with an insertion into VCA0567 (*vxrC*) exhibited a 9-fold reduction in colonization (p<0.0001) [30]. Additionally, Kamp *et al*. found that a strain with a transposon insertion in VCA0565 (*vxrA*) had a disadvantage in fitness (mean fitness value of 0.6) when the bacteria from rabbit cecum fluid was placed into pond water for 48 hours at 30°C [57]. Collectively, these studies suggest that the Vxr genes play important roles in *V*. *cholerae* colonization and environmental dissemination.

Our study revealed that the RR VxrB plays a significant role in colonization and *in vitro* inter-bacterial competition through its ability to regulate expression of T6SS genes. Neither *vxrB* nor any of the *vxrABCDE* operon members show similarity to previously characterized proteins. The *vxr* loci are conserved among the *Vibrio* species *Vibrio parahaemolyticus*, *Vibrio vulnificus*, *Vibrio harveyi*, and *V*. *fischeri*. BLAST analysis revealed that VxrA protein exhibits 67–80%, VxrB 79–84%, VxrC 56–68%, VxrD 58–74%, and VxrE 68–81% sequence similarity to the same proteins in other *Vibrio* species (S2–S4 Figs). We also analyzed the predicted structure and function of the VxrCDE proteins using the protein homology/analogy recognition engine (Phyre) [58]. While VxrC and VxrE could not be modeled with high confidence and sufficient coverage, VxrD exhibited structural similarity to outer membrane protein transport proteins (100% confidence, 90% coverage). These analyses suggest that *vxr* genomic loci are a part of the ancestral *Vibrio* genome, and therefore likely have an evolutionarily conserved role in *Vibrio* biology.

Expression and production of T6SS are tightly regulated at the transcriptional and posttranscriptional levels in a variety of bacterial systems [12, 13, 59]. Environmental signals such as iron limitation, thermoregulation, salinity, envelope stress, indole, and growth on surfaces regulate T6SS expression [59]. In *V*. *cholerae* A1552, the strain used here, T6SS genes are expressed when cell are grown in high-osmolarity and low temperature conditions [50]. A recent study revealed that the *V*. *cholerae* A1552 T6SS genes are part of the competence regulon and their expression is induced when the bacterium grows on chitinous surfaces in a TfoX-, HapR-, and QstR-dependent manner [60]. Our work presented here identified VxrB as a regulator of the T6SS large gene cluster and the two auxiliary clusters. The predicted cognate HK of VxrB, VxrA, does not exhibit similarity to previously characterized sensory domains. The signals that govern expression and activity of VxrAB and how the VxrAB TCS is integrated into the T6SS regulatory network of *V*. *cholerae* are yet to be determined. We determined that while the wild-type strain has a competitive advantage *in vivo* over Δ*vxrB*, neither strain had a competitive advantage over the other *in vitro*. Furthermore, single infection studies showed that Δ*vxrB* had a significant colonization defect compared to wild type, suggesting that VxrB could be involved in competition with normal flora and that Δ*vxrB* could have a reduced fitness in infection environment. These observations also suggest that there may be an *in vivo* signal produced in the infant mouse that triggers T6SS activity and colonization. Our studies thus provided significant new insights into the regulation of T6SS in *V*. *cholerae* and provided further support that the T6SS is critical for *V*. *cholerae* virulence.

## Materials and Methods

### Ethics statement

All animal procedures used were in strict accordance with the NIH *Guide for the Care and Use of Laboratory Animals* [61] and were approved by the UC Santa Cruz Institutional Animal Care and Use Committee (Yildf1206).

### Bacterial strains, plasmids, and culture conditions

The bacterial strains and plasmids used in this study are listed in S1 Table. *Escherichia coli* CC118λpir strains were used for DNA manipulation, and *E*. *coli* S17-1λpir strains were used for conjugation with *V*. *cholerae*. In-frame deletion mutants of *V*. *cholerae* were generated as described earlier [62]. All *V*. *cholerae* and *E*. *coli* strains were grown aerobically, at 30°C and 37°C, respectively, unless otherwise noted. All cultures were grown in Luria-Bertani (LB) broth (1% Tryptone, 0.5% Yeast Extract, 1% NaCl), pH 7.5, unless otherwise stated. LB agar medium contains 1.5% (wt/vol) granulated agar (BD Difco, Franklin Lakes, NJ). AKI medium contains 0.5% NaCl, 0.3% NaHCO_3_, 0.4% Yeast Extract, and 1.5% Peptone, as previously described [63]. Antibiotics were used at the following concentrations: ampicillin 100 μg/ml; rifampicin 100 μg/ml; gentamicin 50 μg/ml; streptomycin 50 μg/ml.

### DNA manipulations

An overlapping PCR method was used to generate in-frame deletion constructs of each RR genes using previously published methods [62]. Briefly, a 500–600 bp 5’ flanking sequence of the gene, including several nucleotides of the coding region, was PCR amplified using del-A and del-B primers. del-C and del-D primers were used to amplify the 3’ region of the gene including 500–600 bp of the downstream flanking sequence. The two PCR products were joined using the splicing overlap extension technique [64, 65] and the resulting PCR product, which lacks 80% of amino acids, was digested with two restriction enzymes and ligated to similarly-digested pGP704*sacB*28 suicide plasmid. Construction of *vxrB* plasmid harboring point mutations were performed using a similar technique [66] with the following alterations: primers containing the new sequence harboring the point mutations were used in place of the del-B and del-C primers. The deletion constructs were sequenced (UC Berkeley DNA Sequencing Facility, Berkeley, CA) and the clones without any undesired mutations were used. The deletion constructs are listed in S1 Table.

### Generation of in-frame deletion mutants and Tn7 complementation strains

The deletion plasmids were maintained in *E*. *coli* CC118λpir. Biparental matings were carried out with the wild type *V*. *cholerae* and an *E*. *coli* S17λpir strain harboring the deletion plasmid. Selection of deletion mutants were done as described [64] and were verified by PCR. The Tn7 complementation *V*. *cholerae* strains were generated by triparental matings with donor *E*. *coli* S17λpir carrying pGP704-Tn7 with gene of interest, helper *E*. *coli* S17λpir harboring pUX-BF13, and *V*. *cholerae* strains. Transconjugants were selected on thiosulfate-citrate-bile salts-sucrose (TCBS) (BD Difco, Franklin Lakes, NJ) agar medium containing gentamicin at 30°C. The Tn7 complementation *V*. *cholerae* strains were verified by PCR.

### Intestinal colonization assay

An *in vivo* competition assay for intestinal colonization was performed as described previously [46]. Briefly, each of the *V*. *cholerae* mutant strains (*lacZ* ^+^) and the fully virulent reference strain (*lacZ* ^-^otherwise wild-type)) were grown to stationary phase at 30°C with aeration in LB broth. Mutant strains and wild-type were mixed at 1:1 ratios in 1x Phosphate Buffered Saline (PBS). The inoculum was plated on LB agar plates containing 5-bromo-4-chloro-3-indoyl-β-D-galactopyranoside (X-gal) to differentiate wild-type and mutant colonies and to determine the input ratios. Approximately, 10^6^–10^7^ cfu were intragastrically administered to groups of 5–7 anesthetized 5-day old CD-1 mice (Charles River Laboratories, Hollister, CA). After 20 hours of incubation, the small intestine was removed, weighed, homogenized, and plated on appropriate selective and differential media to enumerate mutant and wild-type cells recovered and to obtain the output ratios. *In vivo* competitive indices were calculated by dividing the small intestine output ratio by the inoculum input ratio of mutant to wild-type strains. For single strain infections, 10^7^ cfu of each strain, including otherwise wild type (*lacZ* ^-^) strain, were intragastrically administrated to 5-day old CD-1 mice. After 20 hours of incubation, the small intestine was harvested and plated on selective media as previously described above. Statistical analyses for competition infections were performed using Wilcoxon Signed Rank Test. Statistical analyses were performed using Prism 5 software (GraphPad Software, Inc., San Diego, CA) using Wilcoxon Signed Rank Test. P values of <0.05 were determined to be statistically significant.

### Reverse transcription-PCR

RNA was isolated as described below. The reverse transcription reaction to generate cDNA was carried out using the SuperScript III Reverse Transcriptase (Invitrogen, Carlsbad, CA) according to the manufacturer’s instructions at 25°C for 5 min, 50°C for 1 h, and 70°C for 15 min using 1 μg of RNA in a 20 μl final volume. The product was used in a PCR using suitable primers, and RNA without RT treatment was used as a negative control.

### Quantitative real time (qRT) PCR

For qRT-PCR expression analysis, RNA was isolated as described below. cDNA was synthesized using iScript cDNG Synthesis Kit (Bio-Rad, Hercules, CA) from 1 μg of total RNA. Real-time PCR was performed using a Bio-Rad CFX1000 thermal cycler and Bio-RAD CFX96 real-time imager with specific primer pairs (designed within the coding region of the target gene) and SsoAdvanced SYBR green supermix (Bio-Rad, Hercules, CA). Results are from two independent experiments performed in quadruplicate. All samples were normalized to the expression of the housekeeping gene 16S using the Pfaffl method [67]. Relative expression was calculated by normalizing expression at Δ*vxrB* by that of wt. Statistical analysis was performed using two-tailed student’s t test.

### RNA isolation

*V*. *cholerae* cells were grown aerobically overnight in LB at 37°C, then diluted 1:100 in fresh 10 ml AKI media in borosilicate glass test tubes (diameter, 15mm; height, 150 mm) and incubated at 37°C without shaking for 4 hours. After 4 hours, 10 ml cultures were transferred to 125 ml flasks (for maximal aerated growth on an orbital shaker (250 rpm) for 2 hours. Aliquots (2 ml) of the cultures were collected and centrifuged for 2 min at room temperature. The cell pellets were immediately resuspended in 1 ml of TRIzol (Invitrogen, Carlsbad, CA) and stored at -80°C. Total RNA was isolated according to the manufacturer’s instructions. To remove contaminating DNA, total RNA was incubated with RNase-free DNase I (Ambion, Grand Island, NY), and an RNeasy mini kit (Qiagen, Valencia, CA) was used to clean up RNA after DNase digestion. Five micrograms of total RNA was treated with a MICROBExpress Kit (Ambion, Grand Island, NY) to remove ribosomal RNA, and the efficiency was confirmed by Bioanalyzer analysis (Agilent Technologies, Santa Clara, CA). Three biological replicates were generated for each condition.

### cDNA library construction and Illumina HiSeq sequencing

Libraries for RNA-seq were prepared using NEBNext Ultra Directional RNA Library Prep Kit for Illumina (New England Biolabs, Ipswich, MA). Twelve indexed samples were sequenced per single lane using the HiSeq2500 Illumina sequencing platform for 50 bp single reads (UC Davis Genome Center, UC Davis, CA) and subsequently analyzed and visualized via the CLC Genomics Workbench version 7.5 (Qiagen, Valencia, CA). Samples were mapped to the *V*. *cholerae* genome N16961. Differentially regulated genes were identified as those displaying a fold change with an absolute value of 1.5-fold or greater. Statistical significance was determined by Empirical analysis of Digital Gene Expression (edgeR) test where p<0.05 was deemed significant [68].

### Analysis of Hcp production and secretion

*V*. *cholerae* strains were grown to an OD600 of 2.0, and the culture (25 ml) was centrifuged at 20,000 g for 10 min to obtain whole cell pellets. The culture supernatant containing secreted proteins were filtered through 0.22 μ membranes (Millipore, Billerica, Massachusetts) and secreted proteins in the culture supernatant were precipitated with 13% trichloroacetic acid (TCA) overnight at 4°C, pelleted by centrifugation at 47,000 g for 30 min at 4°C, wash with ice cold acetone and resuspended in 1x PBS containing Complete protease inhibitor (Roche, Basel, Switzerland). Bovine serum albumin (BSA, 100 μg/ml) was added to the culture supernatant prior to TCA precipitation as a control. Protein pellets from whole cell were suspended in 2% sodium dodecyl sulfate (SDS) and protein concentrations were estimated using a Pierce BCA protein assay kit (Thermo Scientific, Rockford, IL). Equal amounts of total protein (20 μg) were loaded onto a SDS 13% polyacrylamide gel electrophoresis (SDS-PAGE). Western blot analyses were performed as described [69] using anti-Hcp polyclonal antiserum provided by the Sun Wai [28], anti-CRP (Neoclone Inc., Madison, WI), and anti-BSA (Santa Cruz Biotech, Santa Cruz, CA). OneMinute Western Blot Stripping Buffer (GM Biosciences, Frederick, MD) was used to remove the Hcp antibodies and the same blot was used again to probe for CRP or BSA. These experiments were conducted with at least three biological replicates.

### Bacterial killing assay

Killing assays were performed as described previously [20]. Briefly, bacterial strains were grown overnight on LB plates and resuspended in LB broth containing 340 mM NaCl, as *V*. *cholerae* strain A1552 displayed enhanced interbacterial virulence towards *E*. *coli* under high osmolarity [50]. *V*. *cholerae* and *E*. *coli* MC4100 were mixed at a 10:1 ratio and 25 μl was spotted onto LB agar plates containing 340 mM NaCl and incubated at 37°C for 4 hours. Spots were harvested, serially diluted, and plated onto LB plates containing 50 μg/ml of streptomycin to enumerate surviving *E*. *coli* prey cells.

### *In vitro* competition assay

The following assay was performed similarly as the intestinal colonization assay except no animal models were used. The *V*. *cholerae* mutant strains with wild-type *lacZ* allele (*lacZ* ^+^) and reference strain (*lacZ* ^-^) were grown to stationary phase at 30°C with aeration in LB broth. Mutant strains and wild-type were mixed at 1:1 ratios in 1x PBS. The inoculum was plated on LB agar plates containing X-gal to differentiate colonies formed by the wild-type and mutant strains and to determine the input ratios. The inoculum (50 μl) was spotted on to a LB agar plate and incubated at 37°C. After 20–24 hours of incubation, the 50 μl spots were scraped off the agar plate and resuspended in 1x PBS. The resuspension was serially diluted and plated on appropriate selective and differential media to enumerate mutant and wild type cells recovered and to obtain the output ratios. *In vitro* competitive indices were calculated by dividing the output ratio by the inoculum input ratio of mutant to wild type strains. Statistical analyses were performed using Wilcoxon Signed Rank Test.

## Supporting Information

S1 Fig Confirmation of the predicted operon structure of *vxrABCDE*. RNAseq track reads from wild-type sample. Red and green lines indicate the directionality of the read tracks. Images were prepared by CLC bio version 7.5.1 (Qiagen, Valencia, CA).
(TIF)

S2 Fig Multiple sequence alignment of VxrA. Amino acid sequence alignment of the HK, VxrA, to *V*. *cholerae V52*, *V*. *fischeri MJ11*, *V*. *harveyi ATCC BAA-1116*, *V*. *parahaemolyticus RIMD 2210633*, and *V*.*vulnificus YJ016* using ClustalW. Numbers above the sequence correspond to the amino acid number of each protein.
(TIF)

S3 Fig Multiple sequence alignment of VxrB and VxrC. (A) Amino acid sequence alignment of the RR, VxrB, and (B) VxrC to *V*. *cholerae V52*, *V*. *fischeri MJ11*, *V*. *harveyi ATCC BAA-1116*, *V*. *parahaemolyticus RIMD 2210633*, and *V*. *vulnificus YJ016* using ClustalW. Numbers above the sequence correspond to the amino acid number of each protein.
(TIF)

S4 Fig Multiple sequence alignment of VxrD and VxrE. (A) Amino acid sequence alignment of VxrD, and (B) VxrE to *V*. *cholerae V52*, *V*. *fischeri MJ11*, *V*. *harveyi ATCC BAA-1116*, *V*. *parahaemolyticus RIMD 2210633*, and *V*. *vulnificus YJ016* using ClustalW. Numbers above the sequence correspond to the amino acid number of each protein.
(TIF)

S5 Fig Expression analysis of the T6SS gene clusters. (A) Schematic representation of the major T6SS large gene cluster and auxiliary clusters 1 and 2. (B) RNAseq data showing the coverage of cDNA reads in wild type (wt) and Δ*vxrB* mutant (*vxrB*) over the large cluster and the two auxiliary clusters. Images were prepared by CLC bio version 7.5.1 (Qiagen, Valencia, CA).
(TIF)

S1 Table Bacterial strains and plasmids used in this study. (DOCX)

S2 Table Genes positively regulated by VxrB under AKI conditions. (DOCX)

S3 Table Genes negatively regulated by VxrB under AKI conditions. (DOCX)

## References

[1] Ali M, Lopez AL, You YA, Kim YE, Sah B, Maskery B, et al The global burden of cholera. Bulletin of the World Health Organization. 2012 3 1;90(3):209–18A. 10.2471/BLT.11.093427 22461716 PMC3314202

[2] Kaper JB, Morris JG Jr., Levine MM. Cholera. Clin Microbiol Rev. 1995 1;8(1):48–86. 7704895 10.1128/cmr.8.1.48 PMC172849

[3] DiRita VJ, Parsot C, Jander G, Mekalanos JJ. Regulatory cascade controls virulence in *Vibrio cholerae* . Proc Natl Acad Sci U S A. 1991 6 15;88(12):5403–7. 2052618 10.1073/pnas.88.12.5403 PMC51881

[4] Higgins DE, Nazareno E, DiRita VJ. The virulence gene activator ToxT from *Vibrio cholerae* is a member of the AraC family of transcriptional activators. J Bacteriol. 1992 11;174(21):6974–80. 1400247 10.1128/jb.174.21.6974-6980.1992 PMC207377

[5] Higgins DE, DiRita VJ. Transcriptional control of toxT, a regulatory gene in the ToxR regulon of *Vibrio cholerae* . Mol Microbiol. 1994 10;14(1):17–29. 7830555 10.1111/j.1365-2958.1994.tb01263.x

[6] Hase CC, Mekalanos JJ. TcpP protein is a positive regulator of virulence gene expression in *Vibrio cholerae* . Proc Natl Acad Sci U S A. 1998 1 20;95(2):730–4. 9435261 10.1073/pnas.95.2.730 PMC18489

[7] Krukonis ES, Yu RR, Dirita VJ. The *Vibrio cholerae* ToxR/TcpP/ToxT virulence cascade: distinct roles for two membrane-localized transcriptional activators on a single promoter. Mol Microbiol. 2000 10;38(1):67–84. 11029691 10.1046/j.1365-2958.2000.02111.x

[8] Kovacikova G, Lin W, Skorupski K. *Vibrio cholerae* AphA uses a novel mechanism for virulence gene activation that involves interaction with the LysR-type regulator AphB at the *tcpPH* promoter. Mol Microbiol. 2004 7;53(1):129–42. 15225309 10.1111/j.1365-2958.2004.04121.x

[9] Skorupski K, Taylor RK. A new level in the *Vibrio cholerae* ToxR virulence cascade: AphA is required for transcriptional activation of the *tcpPH* operon. Mol Microbiol. 1999 2;31(3):763–71. 10048021 10.1046/j.1365-2958.1999.01215.x

[10] Kovacikova G, Skorupski K. Regulation of virulence gene expression in *Vibrio cholerae* by quorum sensing: HapR functions at the *aphA* promoter. Mol Microbiol. 2002 11;46(4):1135–47. 12421317 10.1046/j.1365-2958.2002.03229.x

[11] Pukatzki S, Ma AT, Sturtevant D, Krastins B, Sarracino D, Nelson WC, et al Identification of a conserved bacterial protein secretion system in *Vibrio cholerae* using the *Dictyostelium* host model system. Proc Natl Acad Sci U S A. 2006 1 31;103(5):1528–33. 16432199 10.1073/pnas.0510322103 PMC1345711

[12] Ho BT, Dong TG, Mekalanos JJ. A view to a kill: the bacterial type VI secretion system. Cell host & microbe. 2014 1 15;15(1):9–21. 24332978 10.1016/j.chom.2013.11.008 PMC3936019

[13] Silverman JM, Brunet YR, Cascales E, Mougous JD. Structure and regulation of the type VI secretion system. Annu Rev Microbiol. 2012;66:453–72. 10.1146/annurev-micro-121809-151619 22746332 PMC3595004

[14] Pukatzki S, Ma AT, Revel AT, Sturtevant D, Mekalanos JJ. Type VI secretion system translocates a phage tail spike-like protein into target cells where it cross-links actin. Proc Natl Acad Sci U S A. 2007 9 25;104(39):15508–13. 17873062 10.1073/pnas.0706532104 PMC2000545

[15] Dong TG, Ho BT, Yoder-Himes DR, Mekalanos JJ. Identification of T6SS-dependent effector and immunity proteins by Tn-seq in *Vibrio cholerae* . Proc Natl Acad Sci U S A. 2013 2 12;110(7):2623–8. 10.1073/pnas.1222783110 23362380 PMC3574944

[16] Brooks TM, Unterweger D, Bachmann V, Kostiuk B, Pukatzki S. Lytic activity of the *Vibrio cholerae* type VI secretion toxin VgrG-3 is inhibited by the antitoxin TsaB. J Biol Chem. 2013 3 15;288(11):7618–25. 10.1074/jbc.M112.436725 23341465 PMC3597803

[17] Ma AT, McAuley S, Pukatzki S, Mekalanos JJ. Translocation of a *Vibrio cholerae* type VI secretion effector requires bacterial endocytosis by host cells. Cell host & microbe. 2009 3 19;5(3):234–43. 19286133 10.1016/j.chom.2009.02.005 PMC3142922

[18] Russell AB, LeRoux M, Hathazi K, Agnello DM, Ishikawa T, Wiggins PA, et al Diverse type VI secretion phospholipases are functionally plastic antibacterial effectors. Nature. 2013 4 25;496(7446):508–12. 10.1038/nature12074 23552891 PMC3652678

[19] Miyata ST, Kitaoka M, Brooks TM, McAuley SB, Pukatzki S. *Vibrio cholerae* requires the type VI secretion system virulence factor VasX to kill *Dictyostelium discoideum* . Infect Immun. 2011 7;79(7):2941–9. 10.1128/IAI.01266-10 21555399 PMC3191968

[20] Miyata ST, Unterweger D, Rudko SP, Pukatzki S. Dual expression profile of type VI secretion system immunity genes protects pandemic *Vibrio cholerae* . PLoS Pathog. 2013;9(12):e1003752 10.1371/journal.ppat.1003752 24348240 PMC3857813

[21] Unterweger D, Miyata ST, Bachmann V, Brooks TM, Mullins T, Kostiuk B, et al The *Vibrio cholerae* type VI secretion system employs diverse effector modules for intraspecific competition. Nature communications. 2014;5:3549 10.1038/ncomms4549 24686479 PMC3988814

[22] Basler M, Pilhofer M, Henderson GP, Jensen GJ, Mekalanos JJ. Type VI secretion requires a dynamic contractile phage tail-like structure. Nature. 2012 3 8;483(7388):182–6. 10.1038/nature10846 22367545 PMC3527127

[23] Zheng J, Ho B, Mekalanos JJ. Genetic analysis of anti-amoebae and anti-bacterial activities of the type VI secretion system in *Vibrio cholerae* . PloS one. 2011;6(8):e23876 10.1371/journal.pone.0023876 21909372 PMC3166118

[24] Kitaoka M, Miyata ST, Brooks TM, Unterweger D, Pukatzki S. VasH is a transcriptional regulator of the type VI secretion system functional in endemic and pandemic *Vibrio cholerae* . J Bacteriol. 2011 12;193(23):6471–82. 10.1128/JB.05414-11 21949076 PMC3232897

[25] Bernard CS, Brunet YR, Gavioli M, Lloubes R, Cascales E. Regulation of type VI secretion gene clusters by sigma54 and cognate enhancer binding proteins. J Bacteriol. 2011 5;193(9):2158–67. 10.1128/JB.00029-11 21378190 PMC3133059

[26] Dong TG, Mekalanos JJ. Characterization of the RpoN regulon reveals differential regulation of T6SS and new flagellar operons in *Vibrio cholerae* O37 strain V52. Nucleic Acids Res. 2012 9;40(16):7766–75. 10.1093/nar/gks567 22723378 PMC3439928

[27] Zheng J, Shin OS, Cameron DE, Mekalanos JJ. Quorum sensing and a global regulator TsrA control expression of type VI secretion and virulence in *Vibrio cholerae* . Proc Natl Acad Sci U S A. 2010 12 7;107(49):21128–33. 10.1073/pnas.1014998107 21084635 PMC3000250

[28] Ishikawa T, Rompikuntal PK, Lindmark B, Milton DL, Wai SN. Quorum sensing regulation of the two hcp alleles in *Vibrio cholerae* O1 strains. PloS one. 2009;4(8):e6734 10.1371/journal.pone.0006734 19701456 PMC2726435

[29] Ma AT, Mekalanos JJ. *In vivo* actin cross-linking induced by *Vibrio cholerae* type VI secretion system is associated with intestinal inflammation. Proc Natl Acad Sci U S A. 2010 3 2;107(9):4365–70. 10.1073/pnas.0915156107 20150509 PMC2840160

[30] Fu Y, Waldor MK, Mekalanos JJ. Tn-Seq analysis of Vibrio cholerae intestinal colonization reveals a role for T6SS-mediated antibacterial activity in the host. Cell host & microbe. 2013 12 11;14(6):652–63. 24331463 10.1016/j.chom.2013.11.001 PMC3951154

[31] Beier D, Gross R. Regulation of bacterial virulence by two-component systems. Curr Opin Microbiol. 2006 4;9(2):143–52. 16481212 10.1016/j.mib.2006.01.005

[32] Krell T, Lacal J, Busch A, Silva-Jimenez H, Guazzaroni ME, Ramos JL. Bacterial sensor kinases: diversity in the recognition of environmental signals. Annu Rev Microbiol. 2010;64:539–59. 10.1146/annurev.micro.112408.134054 20825354

[33] Galperin MY. Diversity of structure and function of response regulator output domains. Curr Opin Microbiol. 2010 4;13(2):150–9. 10.1016/j.mib.2010.01.005 20226724 PMC3086695

[34] Gao R, Stock AM. Biological insights from structures of two-component proteins. Annu Rev Microbiol. 2009;63:133–54. 10.1146/annurev.micro.091208.073214 19575571 PMC3645274

[35] Laub MT, Goulian M. Specificity in two-component signal transduction pathways. Annual review of genetics. 2007;41:121–45. 18076326 10.1146/annurev.genet.41.042007.170548

[36] Jang J, Jung KT, Park J, Yoo CK, Rhie GE. The *Vibrio cholerae* VarS/VarA two-component system controls the expression of virulence proteins through ToxT regulation. Microbiology. 2011 5;157(Pt 5):1466–73. 10.1099/mic.0.043737-0 21330435

[37] Zhu J, Miller MB, Vance RE, Dziejman M, Bassler BL, Mekalanos JJ. Quorum-sensing regulators control virulence gene expression in *Vibrio cholerae* . Proc Natl Acad Sci U S A. 2002 3 5;99(5):3129–34. 11854465 10.1073/pnas.052694299 PMC122484

[38] Tischler AD, Camilli A. Cyclic diguanylate regulates *Vibrio cholerae* virulence gene expression. Infect Immun. 2005 9;73(9):5873–82. 16113306 10.1128/IAI.73.9.5873-5882.2005 PMC1231145

[39] Tischler AD, Lee SH, Camilli A. The *Vibrio cholerae vieSAB* locus encodes a pathway contributing to cholera toxin production. J Bacteriol. 2002 8;184(15):4104–13. 12107127 10.1128/JB.184.15.4104-4113.2002 PMC135224

[40] Pratt JT, Ismail AM, Camilli A. PhoB regulates both environmental and virulence gene expression in *Vibrio cholerae* . Mol Microbiol. 2010 9;77(6):1595–605. 10.1111/j.1365-2958.2010.07310.x 20659293 PMC2981138

[41] Sengupta N, Paul K, Chowdhury R. The global regulator ArcA modulates expression of virulence factors in *Vibrio cholerae* . Infect Immun. 2003 10;71(10):5583–9. 14500477 10.1128/IAI.71.10.5583-5589.2003 PMC201065

[42] Bilecen K, Fong JC, Cheng A, Jones CJ, Zamorano-Sanchez D, Yildiz FH. Polymyxin B Resistance and biofilm formation in *Vibrio cholerae* is controlled by the response regulator CarR. Infect Immun. 2015 1 12. 10.1128/IAI.02700-14 PMC4333464 25583523

[43] Butler SM, Camilli A. Both chemotaxis and net motility greatly influence the infectivity of *Vibrio cholerae* . Proc Natl Acad Sci U S A. 2004 4 6;101(14):5018–23. 15037750 10.1073/pnas.0308052101 PMC387366

[44] Correa NE, Lauriano CM, McGee R, Klose KE. Phosphorylation of the flagellar regulatory protein FlrC is necessary for *Vibrio cholerae* motility and enhanced colonization. Mol Microbiol. 2000 2;35(4):743–55. 10692152 10.1046/j.1365-2958.2000.01745.x

[45] Butler SM, Camilli A. Going against the grain: chemotaxis and infection in *Vibrio cholerae* . Nature reviews Microbiology. 2005 8;3(8):611–20. 16012515 10.1038/nrmicro1207 PMC2799996

[46] Taylor RK, Miller VL, Furlong DB, Mekalanos JJ. Use of *phoA* gene fusions to identify a pilus colonization factor coordinately regulated with cholera toxin. Proc Natl Acad Sci U S A. 1987 5;84(9):2833–7. 2883655 10.1073/pnas.84.9.2833 PMC304754

[47] Galperin MY. Structural classification of bacterial response regulators: diversity of output domains and domain combinations. J Bacteriol. 2006 6;188(12):4169–82. 16740923 10.1128/JB.01887-05 PMC1482966

[48] Delgado J, Forst S, Harlocker S, Inouye M. Identification of a phosphorylation site and functional analysis of conserved aspartic acid residues of OmpR, a transcriptional activator for *ompF* and *ompC* in *Escherichia coli* . Mol Microbiol. 1993 12;10(5):1037–47. 7934854 10.1111/j.1365-2958.1993.tb00974.x

[49] Ma S, Selvaraj U, Ohman DE, Quarless R, Hassett DJ, Wozniak DJ. Phosphorylation-independent activity of the response regulators AlgB and AlgR in promoting alginate biosynthesis in mucoid *Pseudomonas aeruginosa* . J Bacteriol. 1998 2;180(4):956–68. 9473053 10.1128/jb.180.4.956-968.1998 PMC106978

[50] Ishikawa T, Sabharwal D, Broms J, Milton DL, Sjostedt A, Uhlin BE, et al Pathoadaptive conditional regulation of the type VI secretion system in *Vibrio cholerae* O1 strains. Infect Immun. 2012 2;80(2):575–84. 10.1128/IAI.05510-11 22083711 PMC3264300

[51] Hussa EA, O'Shea TM , Darnell CL, Ruby EG, Visick KL. Two-component response regulators of Vibrio fischeri: identification, mutagenesis, and characterization. J Bacteriol. 2007 8;189(16):5825–38. 10.1128/JB.00242-07 PMC1952042 17586650

[52] Kobayashi K, Ogura M, Yamaguchi H, Yoshida K, Ogasawara N, Tanaka T, et al Comprehensive DNA microarray analysis of *Bacillus subtilis* two-component regulatory systems. J Bacteriol. 2001 12;183(24):7365–70. 11717295 10.1128/JB.183.24.7365-7370.2001 PMC95585

[53] Lange R, Wagner C, de Saizieu A, Flint N, Molnos J, Stieger M, et al Domain organization and molecular characterization of 13 two-component systems identified by genome sequencing of *Streptococcus pneumoniae* . Gene. 1999 9 3;237(1):223–34. 10524254 10.1016/s0378-1119(99)00266-8

[54] Throup JP, Koretke KK, Bryant AP, Ingraham KA, Chalker AF, Ge Y, et al A genomic analysis of two-component signal transduction in *Streptococcus pneumoniae* . Mol Microbiol. 2000 2;35(3):566–76. 10672179 10.1046/j.1365-2958.2000.01725.x

[55] Yamamoto K, Hirao K, Oshima T, Aiba H, Utsumi R, Ishihama A. Functional characterization in vitro of all two-component signal transduction systems from *Escherichia coli* . J Biol Chem. 2005 1 14;280(2):1448–56. 15522865 10.1074/jbc.M410104200

[56] Mandlik A, Livny J, Robins WP, Ritchie JM, Mekalanos JJ, Waldor MK. RNA-Seq-based monitoring of infection-linked changes in *Vibrio cholerae* gene expression. Cell host & microbe. 2011 8 18;10(2):165–74. 21843873 10.1016/j.chom.2011.07.007 PMC3166260

[57] Kamp HD, Patimalla-Dipali B, Lazinski DW, Wallace-Gadsden F, Camilli A. Gene fitness landscapes of *Vibrio cholerae* at important stages of its life cycle. PLoS Pathog. 2013;9(12):e1003800 10.1371/journal.ppat.1003800 24385900 PMC3873450

[58] Kelley LA, Sternberg MJ. Protein structure prediction on the Web: a case study using the Phyre server. Nature protocols. 2009;4(3):363–71. 10.1038/nprot.2009.2 19247286

[59] Bernard CS, Brunet YR, Gueguen E, Cascales E. Nooks and crannies in type VI secretion regulation. J Bacteriol. 2010 8;192(15):3850–60. 10.1128/JB.00370-10 20511495 PMC2916374

[60] Borgeaud S, Metzger LC, Scrignari T, Blokesch M. Bacterial evolution. The type VI secretion system of *Vibrio cholerae* fosters horizontal gene transfer. Science. 2015 1 2;347(6217):63–7. 10.1126/science.1260064 25554784

[61] National Research Council (U.S.). Committee for the Update of the Guide for the Care and Use of Laboratory Animals, Institute for *Laboratory* Animal Research (U.S.), National Academies Press (U.S.). Guide for the care and use of laboratory animals. 8th ed Washington, D.C.: National Academies Press; 2011 xxv, 220 p. p.

[62] Lim B, Beyhan S, Meir J, Yildiz FH. Cyclic-diGMP signal transduction systems in *Vibrio cholerae*: modulation of rugosity and biofilm formation. Mol Microbiol. 2006 4;60(2):331–48. 16573684 10.1111/j.1365-2958.2006.05106.x

[63] Iwanaga M, Yamamoto K, Higa N, Ichinose Y, Nakasone N, Tanabe M. Culture conditions for stimulating cholera toxin production by *Vibrio cholerae* O1 El Tor. Microbiology and immunology. 1986;30(11):1075–83. 3543624 10.1111/j.1348-0421.1986.tb03037.x

[64] Fong JC, Yildiz FH. The *rbmBCDEF* gene cluster modulates development of rugose colony morphology and biofilm formation in *Vibrio cholerae* . J Bacteriol. 2007 3;189(6):2319–30. 17220218 10.1128/JB.01569-06 PMC1899372

[65] Lefebvre B, Formstecher P, Lefebvre P. Improvement of the gene splicing overlap (SOE) method. BioTechniques. 1995 8;19(2):186–8. 8527132

[66] Beyhan S, Bilecen K, Salama SR, Casper-Lindley C, Yildiz FH. Regulation of rugosity and biofilm formation in *Vibrio cholerae*: comparison of VpsT and VpsR regulons and epistasis analysis of *vpsT*, *vpsR*, and *hapR* . J Bacteriol. 2007 1;189(2):388–402. 17071756 10.1128/JB.00981-06 PMC1797413

[67] Pfaffl MW. A new mathematical model for relative quantification in real-time RT-PCR. Nucleic Acids Res. 2001 5 1;29(9):e45 11328886 10.1093/nar/29.9.e45 PMC55695

[68] Robinson MD, McCarthy DJ, Smyth GK. edgeR: a Bioconductor package for differential expression analysis of digital gene expression data. Bioinformatics. 2010 1 1;26(1):139–40. 10.1093/bioinformatics/btp616 19910308 PMC2796818

[69] Berk V, Fong JC, Dempsey GT, Develioglu ON, Zhuang X, Liphardt J, et al Molecular architecture and assembly principles of *Vibrio cholerae* biofilms. Science. 2012 7 13;337(6091):236–9. 10.1126/science.1222981 22798614 PMC3513368

