# Supplementary figures and images for: Vibrio cholerae Response Regulator VxrB Controls Colonization and Regulates the Type VI Secretion System
Source: PLoS Pathog. 2015 May 22;11(5):e1004933. doi: 10.1371/journal.ppat.1004933 (PMC4441509; doi:10.1371/journal.ppat.1004933)

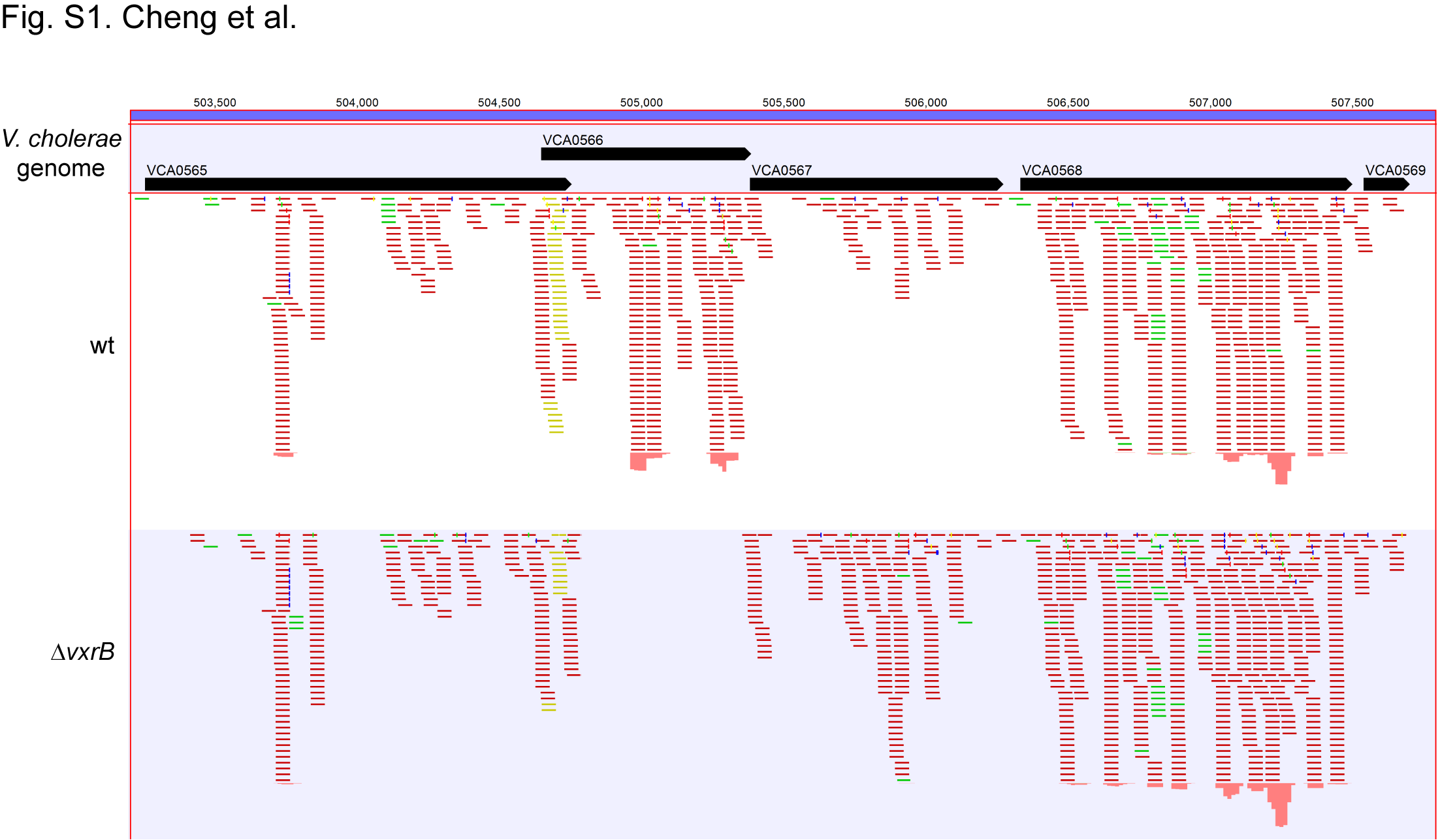

Supplement: S1 Fig — RNAseq track reads from wild-type sample. Red and green lines indicate the directionality of the read tracks. Images were prepared by CLC bio version 7.5.1 (Qiagen, Valencia, CA). (TIF) [file ppat.1004933.s001.tif]

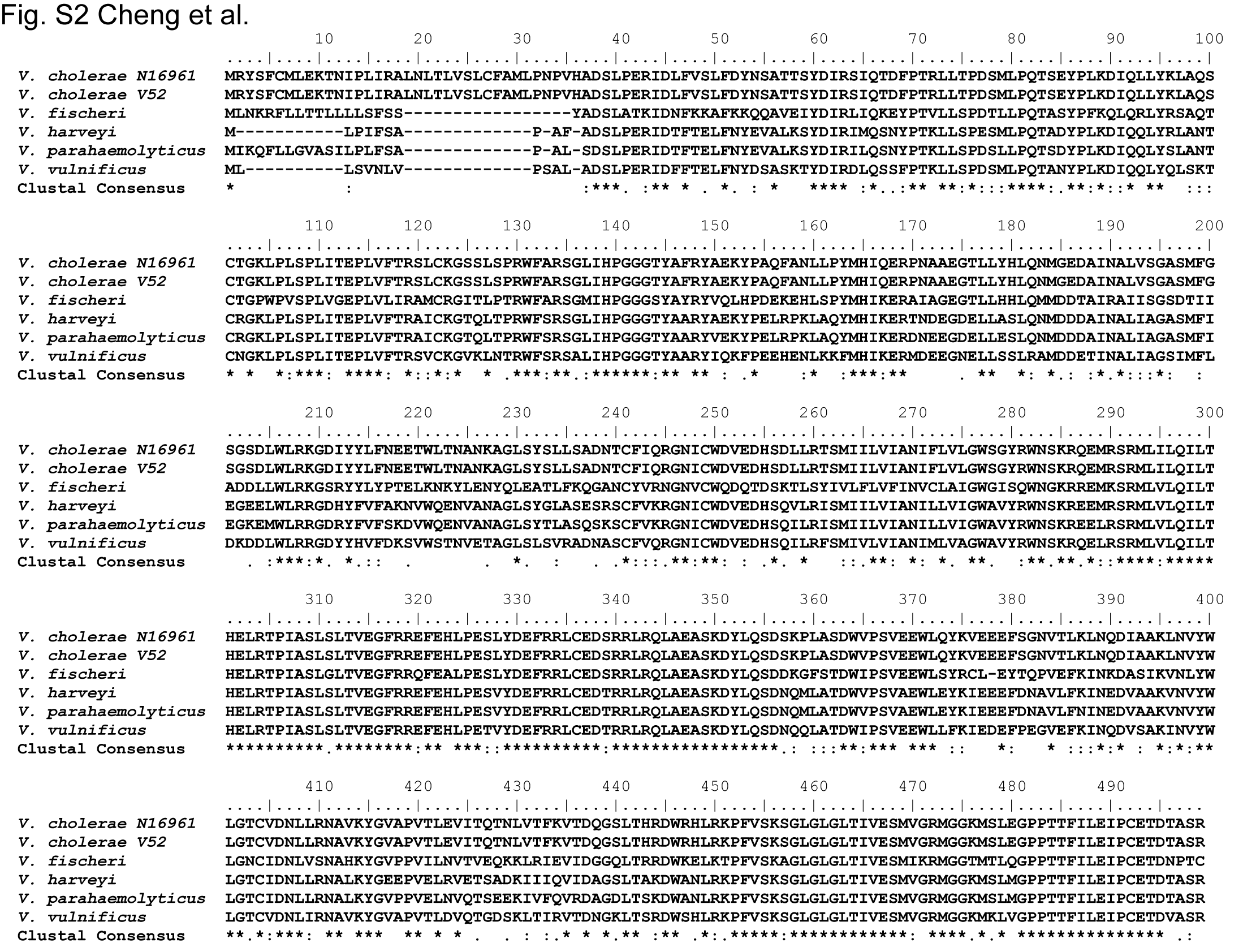

Supplement: S2 Fig — Amino acid sequence alignment of the HK, VxrA, to V. cholerae V52, V. fischeri MJ11, V. harveyi ATCC BAA-1116, V. parahaemolyticus RIMD 2210633, and V.vulnificus YJ016 using ClustalW. Numbers above the sequence correspond to the amino acid number of each protein. (TIF) [file ppat.1004933.s002.tif]

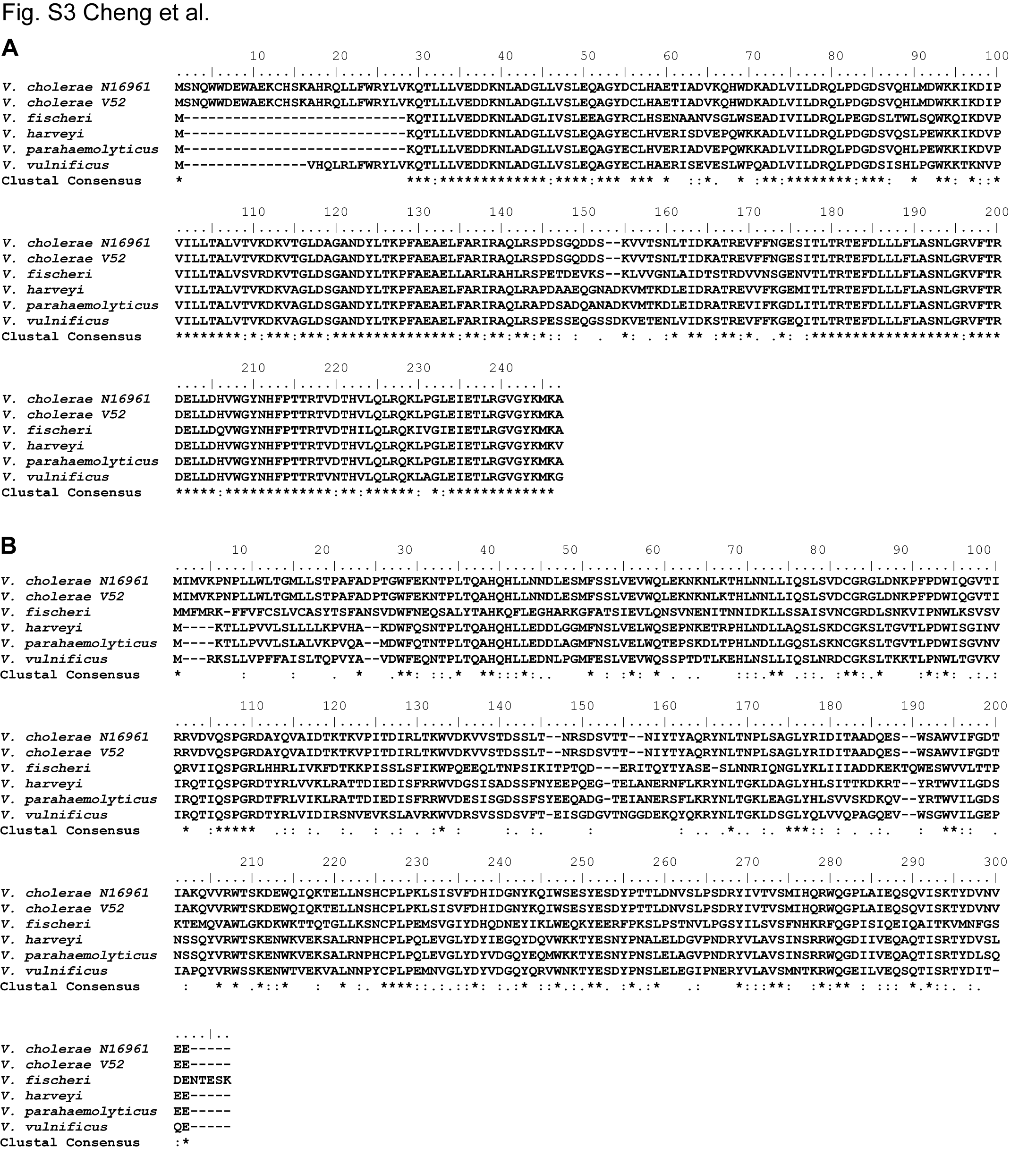

Supplement: S3 Fig — (A) Amino acid sequence alignment of the RR, VxrB, and (B) VxrC to V. cholerae V52, V. fischeri MJ11, V. harveyi ATCC BAA-1116, V. parahaemolyticus RIMD 2210633, and V. vulnificus YJ016 using ClustalW. Numbers above the sequence correspond to the amino acid number of each protein. (TIF) [file ppat.1004933.s003.tif]

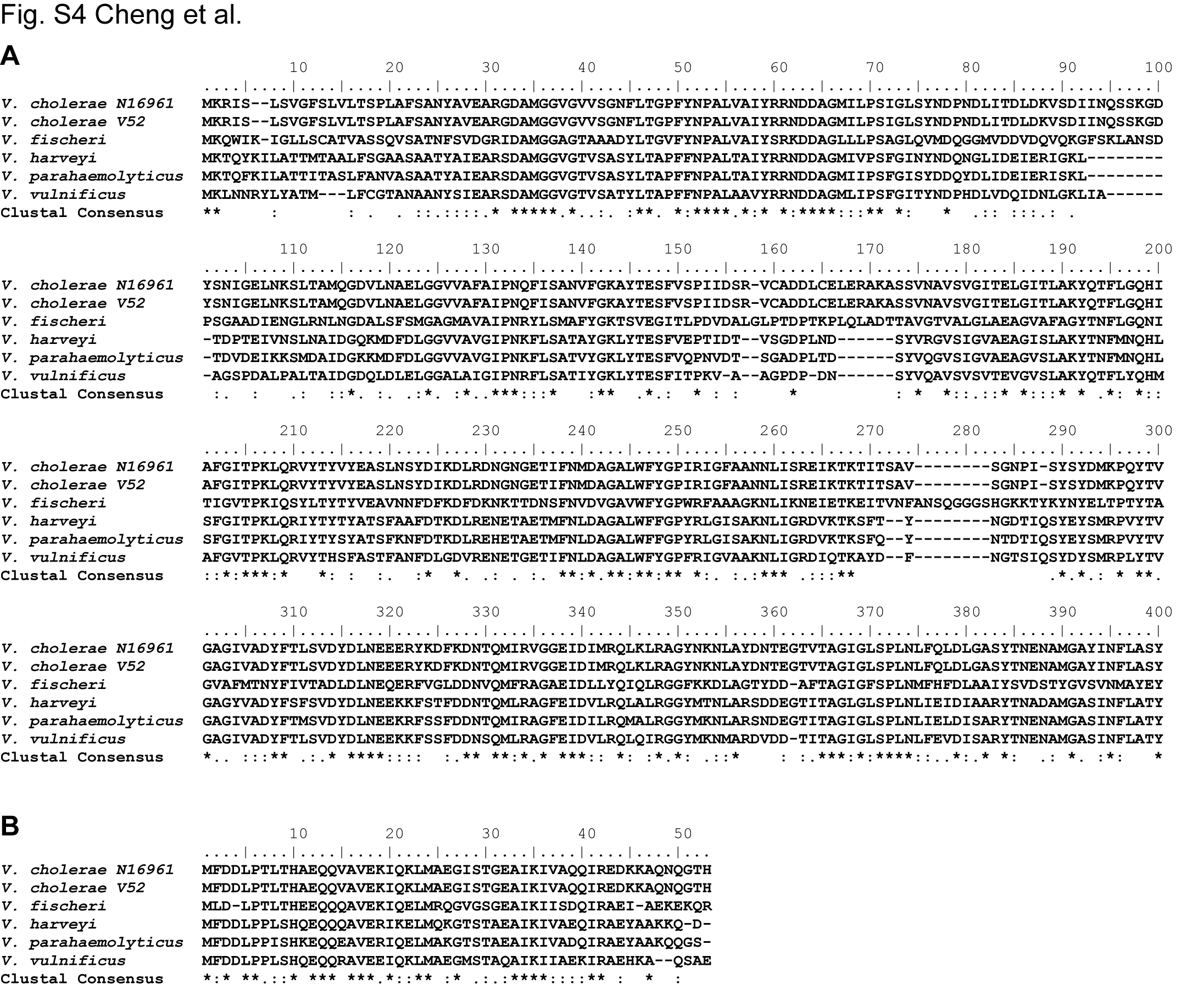

Supplement: S4 Fig — (A) Amino acid sequence alignment of VxrD, and (B) VxrE to V. cholerae V52, V. fischeri MJ11, V. harveyi ATCC BAA-1116, V. parahaemolyticus RIMD 2210633, and V. vulnificus YJ016 using ClustalW. Numbers above the sequence correspond to the amino acid number of each protein. (TIF) [file ppat.1004933.s004.tif]

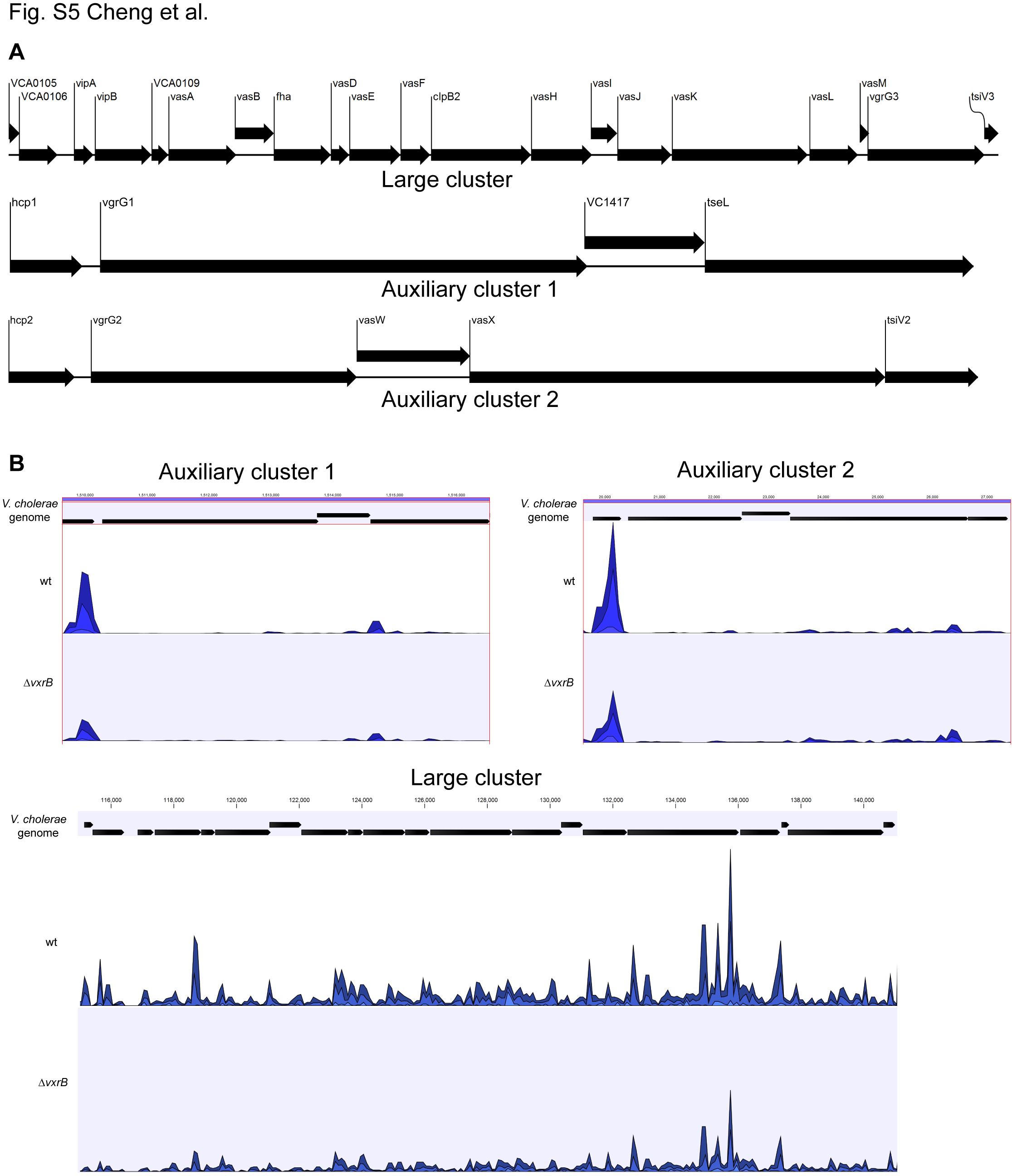

Supplement: S5 Fig — (A) Schematic representation of the major T6SS large gene cluster and auxiliary clusters 1 and 2. (B) RNAseq data showing the coverage of cDNA reads in wild type (wt) and ΔvxrB mutant (vxrB) over the large cluster and the two auxiliary clusters. Images were prepared by CLC bio version 7.5.1 (Qiagen, Valencia, CA). (TIF) [file ppat.1004933.s005.tif]
