# Supplementary material for: Vibrio cholerae Response Regulator VxrB Controls Colonization and Regulates the Type VI Secretion System
Source: PLoS Pathog. 2015 May 22;11(5):e1004933. doi: 10.1371/journal.ppat.1004933 (PMC4441509; doi:10.1371/journal.ppat.1004933)
Supplement: S1 Table — (DOCX) [file ppat.1004933.s006.docx]

**S1 Table. Bacterial strains and plasmids used in this study.**

| Strain or plasmid | | Relevant genotype | Source |
| --- | --- | --- | --- |
| *E. coli* strains | | | |
|  | CC118λ*pir* | Δ(*ara*-*leu*) *araD* Δ*lacX74* *galE* *galK* *phoA20* *thi*-*1* *rpsE* *rpoB* *argE*(*Am*) *recA1* λ*pir* | [[1](#_ENREF_1)] |
|  | S17-1λ*pir* | Tp^r^ Sm^r^ *recA thi pro* r_K_^-^ m_K_^+^ RP4::2-Tc::MuKm Tn*7* λ*pir* | [[2](#_ENREF_2)] |
|  | MC4100 | F^-^ *araD139* Δ(*argF-lac*)U169 *rpsL150*(*st*r*^r^*) *relA1* *deoC1* *rbsR* *fthD5301* *fruA25* λ^-^ | Ottemann lab |
| *V. cholerae* strains | | | |
|  | FY_VC_0001 | *Vibrio cholerae* O1 El Tor A1552, wild type, Rif^r^ | [[3](#_ENREF_3)] |
|  | FY_VC_0003 | Δ*lacZ*, Rif^r^ | [[4](#_ENREF_4)] |
|  | FY_VC_8327 | ΔVC0396 (*qstR*) | This study |
|  | FY_VC_2272 | ΔVC0665 (*vpsR*) | [[5](#_ENREF_5)] |
|  | FY_VC_8197 | ΔVC0693 | This study |
|  | FY_VC_8162 | ΔVC0719 (*phoB*) | This study |
|  | FY_VC_8074 | ΔVC0790 | This study |
|  | FY_VC_0192 | ΔVC1021 (*luxO*) | This study |
|  | FY_VC_8480 | ΔVC1050 | This study |
|  | FY_VC_8329 | ΔVC1081 | This study |
|  | FY_VC_8180 | ΔVC1082 | This study |
|  | FY_VC_1936 | ΔVC1086 | This study |
|  | FY_VC_2760 | ΔVC1087 | This study |
|  | FY_VC_0692 | ΔVC1155 | This study |
|  | FY_VC_8474 | ΔVC1213 (*varA*) | This study |
|  | FY_VC_4379 | ΔVC1277 | This study |
|  | FY_VC_3282 | ΔVC1320 (*carR*) | [[6](#_ENREF_6)] |
|  | FY_VC_8331 | ΔVC1348 | This study |
|  | FY_VC_7998 | ΔVC1522 | This study |
|  | FY_VC_8482 | ΔVC1604 | This study |
|  | FY_VC_8164 | ΔVC1638 | This study |
|  | FY_VC_8706 | ΔVC1651 (*vieB*) | This study |
|  | FY_VC_0516 | ΔVC1652 (*vieA*) | [[7](#_ENREF_7)] |
|  | FY_VC_8166 | ΔVC1719 (*torR*) | This study |
|  | FY_VC_8243 | ΔVC1926 (*dct-D1*) | This study |
|  | FY_VC_6286 | ΔVC2135 (*flrC*) | This study |
|  | FY_VC_8756 | ΔVC2692 (*cpxR*) | This study |
|  | FY_VC_8708 | ΔVC2702 (*cbrR*) | This study |
|  | FY_VC_8179 | ΔVC2714 (*ompR*) | This study |
|  | FY_VC_6289 | ΔVC2749 (*ntrC*) | This study |
|  | FY_VC_8245 | ΔVCA0142 (*dct-D2*) | This study |
|  | FY_VC_2315 | ΔVCA0210 | This study |
|  | FY_VC_8194 | ΔVCA0239 | This study |
|  | FY_VC_8148 | ΔVCA0256 | This study |
|  | FY_VC_8150 | ΔVCA0532 | This study |
|  | FY_VC_9332 | ΔVCA0565 (*vxrA*) | This study |
|  | FY_VC_8758 | ΔVCA0566 (*vxrB*) | This study |
|  | FY_VC_9369 | ΔVCA0567 (*vxrC*) | This study |
|  | FY_VC_9417 | ΔVCA0568 (*vxrD*) | This study |
|  | FY_VC_9394 | ΔVCA0569 (*vxrE*) | This study |
|  | FY_VC_8478 | ΔVCA0682 (*uhpA*) | This study |
|  | FY_VC_7969 | ΔVCA0704 (*pgtA*) | This study |
|  | FY_VC_8209 | ΔVCA0850 | This study |
|  | FY_VC_0099 | ΔVCA0952 (*vpsT*) | [[4](#_ENREF_4)] |
|  | FY_VC_8841 | ΔVCA1086 | This study |
|  | FY_VC_8154 | ΔVCA1105 | This study |
|  | FY_VC_9460 | *vxrB*::D78A | This study |
|  | FY_VC_9462 | *vxrB*::D78E | This study |
|  | FY_VC_9469 | SΔ*vxrB*-Tn7::*vxrB* | This study |
|  | FY_VC_9952 | Δ*vxrB*Δ*hcp1*Δ*hcp2* | This study |
|  | FY_VC_9569 | ΔVC1415ΔVCA0017 (Δ*hcp1*Δ*hcp2*) | This study |
|  | FY_VC_9735 | Δ*hcp1*Δ*hcp2*-Tn7-*hcp1* | This study |
|  | FY_VC_9737 | Δ*hcp1*Δ*hcp2*-Tn7-*hcp2* | This study |
|  | FY_VC_9562 | ΔVCA0117 (Δ*vasH*) | This study |
|  |  | ΔVCA0123 (Δ*vgrG3*) | [[8](#_ENREF_8)] |
|  |  |  |  |
| Plasmids | | | |
|  | pGP704*sacB*28 | pGP704 derivative, *mob*/*oriT* *sacB*, Ap^r^ | [[4](#_ENREF_4)] |
|  | pFY-0484 | pGP704-*sacB28*::ΔVC0396, Ap^r^ | This study |
|  | pFY-1656 | pGP704-*sacB28*::ΔVC0693, Ap^r^ | This study |
|  | pFY-1402 | pGP704-*sacB28*::ΔVC0719, Ap^r^ | This study |
|  | pFY-1826 | pGP704-*sacB28*::ΔVC0790, Ap^r^ | This study |
|  | pFY-0008 | pGP704-*sacB28*::ΔVC1021, Ap^r^ | This study |
|  | pFY-1840 | pGP704-*sacB28*::ΔVC1050, Ap^r^ | This study |
|  | pFY-0120 | pGP704-*sacB28*::ΔVC1081, Ap^r^ | This study |
|  | pFY-1651 | pGP704-*sacB28*::ΔVC1082, Ap^r^ | This study |
|  | pFY-1748 | pGP704-*sacB28*::ΔVC1086, Ap^r^ | This study |
|  | pFY-0562 | pGP704-*sacB28*::ΔVC1087, Ap^r^ | This study |
|  | pFY-0124 | pGP704-*sacB28*::ΔVC1155, Ap^r^ | This study |
|  | pFY-1829 | pGP704-*sacB28*::ΔVC1213, Ap^r^ | This study |
|  | pFY-0766 | pGP704-*sacB28*::ΔVC1277, Ap^r^ | This study |
|  | pFY-0116 | pGP704-*sacB28*::ΔVC1348, Ap^r^ | This study |
|  | pFY-1345 | pGP704-*sacB28*::ΔVC1522, Ap^r^ | This study |
|  | pFY-1844 | pGP704-*sacB28*::ΔVC1604, Ap^r^ | This study |
|  | pFY-1405 | pGP704-*sacB28*::ΔVC1638, Ap^r^ | This study |
|  | pFY-1847 | pGP704-*sacB28*::ΔVC1651, Ap^r^ | This study |
|  | pFY-1655 | pGP704-*sacB28*::ΔVC1719, Ap^r^ | This study |
|  | pFY-1820 | pGP704-*sacB28*::ΔVC1926, Ap^r^ | This study |
|  | pFY-1077 | pGP704-*sacB28*::ΔVC2135, Ap^r^ | This study |
|  | pFY-1917 | pGP704-*sacB28*::ΔVC2692, Ap^r^ | This study |
|  | pFY-1898 | pGP704-*sacB28*::ΔVC2702, Ap^r^ | This study |
|  | pFY-1411 | pGP704-*sacB28*::ΔVC2714, Ap^r^ | This study |
|  | pFY-1073 | pGP704-*sacB28*::ΔVC2749, Ap^r^ | This study |
|  | pFY-1818 | pGP704-*sacB28*::ΔVCA0142, Ap^r^ | This study |
|  | pFY-0471 | pGP704-*sacB28*::ΔVCA0210, Ap^r^ | This study |
|  | pFY-0115 | pGP704-*sacB28*::ΔVCA0239, Ap^r^ | This study |
|  | pFY-1660 | pGP704-*sacB28*::ΔVCA0256, Ap^r^ | This study |
|  | pFY-1338 | pGP704-*sacB28*::ΔVCA0532, Ap^r^ | This study |
|  | pFY-2117 | pGP704-*sacB28*::ΔVCA0565, Ap^r^ | This study |
|  | pFY-1914 | pGP704-*sacB28*::ΔVCA0566, Ap^r^ | This study |
|  | pFY-2119 | pGP704-*sacB28*::ΔVCA0567, Ap^r^ | This study |
|  | pFY-3075 | pGP704-*sacB28*::ΔVCA0568, Ap^r^ | This study |
|  | pFY-2126 | pGP704-*sacB28*::ΔVCA0569, Ap^r^ | This study |
|  | pFY-1832 | pGP704-*sacB28*::ΔVCA0682, Ap^r^ | This study |
|  | pFY-1311 | pGP704-*sacB28*::ΔVCA0704, Ap^r^ | This study |
|  | pFY-440 | pGP704-*sacB28*::ΔVCA0850, Ap^r^ | This study |
|  | pFY-1986 | pGP704-*sacB28*::ΔVCA1086, Ap^r^ | This study |
|  | pFY-1658 | pGP704-*sacB28*::ΔVCA1105, Ap^r^ | This study |
|  | pFY-3563 | pGP704-*sacB28*::ΔVCA0566 (D78A), Ap^r^ | This study |
|  | pFY-3565 | pGP704-*sacB28*::ΔVCA0566 (D78E), Ap^r^ | This study |
|  | pFY-1041 | pGP704-*sacB28*::ΔVC1415, Ap^r^ | This study |
|  | pFY-1042 | pGP704-*sacB28*::ΔVCA0017, Ap^r^ | This study |
|  | pFY-1043 | pGP704-*sacB28*::ΔVCA0117, Ap^r^ | This study |
|  | pFY-3573 | pGP704-Tn7-*vxrB*, Gm^r^, Ap^r^ | This study |
|  | pFY-4154 | pGP704-Tn7-*hcp1*, Gm^r^, Ap^r^ | This study |
|  | pFY-4156 | pGP704-Tn7-*hcp2*, Gm^r^, Ap^r^ | This study |
|  | pUX-BF13 | oriR6K helper plasmid, mob/oriT, provides the Tn7 transposition function in trans, Ap^r^ | [[9](#_ENREF_9)] |
|  | pMCM11 | pGP704::mTn*7*-*gfp*, Gm^r^ Ap^r^ | M. Miller and G. Schoolnik |

**References**

1. Herrero M, de Lorenzo V, Timmis KN. Transposon vectors containing non-antibiotic resistance selection markers for cloning and stable chromosomal insertion of foreign genes in gram-negative bacteria. J Bacteriol. 1990 Nov;172(11):6557-67.

2. de Lorenzo V, Timmis KN. Analysis and construction of stable phenotypes in gram-negative bacteria with Tn5- and Tn10-derived minitransposons. Methods in enzymology. 1994;235:386-405.

3. Yildiz FH, Liu XS, Heydorn A, Schoolnik GK. Molecular analysis of rugosity in a *Vibrio* *cholerae* O1 El Tor phase variant. Mol Microbiol. 2004 Jul;53(2):497-515.

4. Casper-Lindley C, Yildiz FH. VpsT is a transcriptional regulator required for expression of vps biosynthesis genes and the development of rugose colonial morphology in *Vibrio* *cholerae* O1 El Tor. J Bacteriol. 2004 Mar;186(5):1574-8.

5. Yildiz FH, Dolganov NA, Schoolnik GK. VpsR, a Member of the Response Regulators of the Two-Component Regulatory Systems, Is Required for Expression of *vps* Biosynthesis Genes and EPS(ETr)-Associated Phenotypes in *Vibrio* *cholerae* O1 El Tor. J Bacteriol. 2001 Mar;183(5):1716-26.

6. Bilecen K, Yildiz FH. Identification of a calcium-controlled negative regulatory system affecting *Vibrio* *cholerae* biofilm formation. Environ Microbiol. 2009 Aug;11(8):2015-29.

7. Beyhan S, Tischler AD, Camilli A, Yildiz FH. Differences in gene expression between the classical and El Tor biotypes of *Vibrio* *cholerae* O1. Infect Immun. 2006 Jun;74(6):3633-42.

8. Ishikawa T, Sabharwal D, Broms J, Milton DL, Sjostedt A, Uhlin BE, et al. Pathoadaptive conditional regulation of the type VI secretion system in *Vibrio* *cholerae* O1 strains. Infect Immun. 2012 Feb;80(2):575-84.

9. Bao Y, Lies DP, Fu H, Roberts GP. An improved Tn7-based system for the single-copy insertion of cloned genes into chromosomes of gram-negative bacteria. Gene. 1991 Dec 20;109(1):167-8.
