# Supplementary material for: Vibrio cholerae Response Regulator VxrB Controls Colonization and Regulates the Type VI Secretion System
Source: PLoS Pathog. 2015 May 22;11(5):e1004933. doi: 10.1371/journal.ppat.1004933 (PMC4441509; doi:10.1371/journal.ppat.1004933)
Supplement: S2 Table — (DOCX) [file ppat.1004933.s007.docx]

**S2 Table. Genes positively regulated by VxrB under AKI conditions**

| ORF ID^a^ | Gene | Fold Down Regulation^b^ | p-value | Predicted function |
| --- | --- | --- | --- | --- |
| VC2662 |  | -6.69 | 0.00000 | Polyhydroxyalkanoic acid synthase |
| VCA0376 |  | -6.62 | 0.02888 | Hypothetical protein |
| VCA0105 |  | -4.44 | 0.00000 | PAAR motif |
| VCA0125 |  | -4.13 | 0.00000 | Prokaryotic membrane lipoprotein lipid attachment site profile |
| VC0483 |  | -3.83 | 0.00000 | Oxidative stress defense protein |
| VCA0035 |  | -3.60 | 0.00000 | Phosphatidylglycerophosphatase B |
| VC1454 | *rstA1* | -3.55 | 0.02341 | Putative phage replication protein |
| VCA0451 |  | -3.55 | 0.02444 | Hypothetical protein |
| VC0157 |  | -3.28 | 0.00000 | Alkaline serine protease |
| VC1160 |  | -3.26 | 0.00000 | Glutathione synthase/glutaminyl transferase |
| VCA0271 |  | -3.19 | 0.00000 | Lambda repressor-like, DNA-binding |
| VCA0484 |  | -3.18 | 0.00249 | Hypothetical protein |
| VC1415 | *hcp-1* | -3.02 | 0.00000 | T6SS |
| VCA0106 |  | -2.97 | 0.00000 | T6SS |
| VCA0017 | *hcp-2* | -2.82 | 0.00000 | T6SS |
| VCA0026 |  | -2.73 | 0.00000 | Hypothetical protein |
| VCA0112 | *fha* | -2.72 | 0.00000 | T6SS |
| VC1484 | *rmf* | -2.70 | 0.00000 | Ribosome modulation factor |
| VC1962 | *nlpE* | -2.64 | 0.00000 | Copper resistance lipoprotein |
| VCA0140 |  | -2.62 | 0.00000 | Spindolin-related protein |
| VCA0229 | *vctG* | -2.42 | 0.01903 | Iron(III) ABC transporter |
| VCA0109 |  | -2.39 | 0.00007 | T6SS |
| VC1162 |  | -2.34 | 0.00000 | Peptidase aspartic lipoprotein |
| VC2612 |  | -2.31 | 0.01225 | YehU-like superfamily |
| VCA0113 | *vasD* | -2.29 | 0.00000 | T6SS |
| VC1161 |  | -2.28 | 0.00000 | Gonadoliberin III-related protein |
| VCA0845 |  | -2.24 | 0.00000 | Hypothetical protein |
| VC2518 |  | -2.17 | 0.00000 | ABC-type transport resistance to organic solvents |
| VCA0122 | *vasM* | -2.17 | 0.02985 | T6SS |
| VCA0846 |  | -2.16 | 0.00000 | Putative threonine efflux protein |
| VCA0365 |  | -2.14 | 0.00119 | Hypothetical protein |
| VCA0677 | *napD* | -2.13 | 0.00002 | Nitrate reductase assembly |
| VC0255 | *rfbT* | -2.12 | 0.01445 | Serotype conversion |
| VCA0107 | *vipA* | -2.11 | 0.00113 | T6SS |
| VCA0676 | *napF* | -2.11 | 0.00017 | Iron-sulfur cluster-binding protein |
| VC2386 |  | -2.10 | 0.04729 | ATP-binding protein in DNA repair |
| VC2213 | *ompA* | -2.08 | 0.00000 | Outer membrane protein A |
| VC0248 |  | -2.07 | 0.03509 | Acyl carrier protein |
| VC1947 |  | -2.07 | 0.00250 | LysR family transcriptional regulator |
| VCA0108 | *vipB* | -2.05 | 0.00000 | T6SS |
| VCA0116 | *clpB-2* | -2.05 | 0.00000 | T6SS |
| VCA0917 |  | -2.05 | 0.00000 | TetR family transcriptional regulator |
| VCA0111 | *vasB* | -1.99 | 0.00001 | T6SS |
| VCA0915 | *hutD* | -1.97 | 0.00000 | Hemin importer |
| VCA0121 | *vasL* | -1.96 | 0.00000 | T6SS |
| VCA0120 | *vasK* | -1.95 | 0.00000 | T6SS |
| VCA0114 | *vasE* | -1.89 | 0.00000 | T6SS |
| VC0687 | *cstA* | -1.87 | 0.00000 | Carbon starvation protein A |
| VCA0117 | *vasH* | -1.87 | 0.00000 | T6SS |
| VCA0115 | *vasF* | -1.85 | 0.00001 | T6SS |
| VC2076 |  | -1.81 | 0.01176 | FeoC transcriptional regulator |
| VCA0119 | *vasJ* | -1.77 | 0.00000 | T6SS |
| VCA0426 |  | -1.76 | 0.00828 | Iron-sulfur protein |
| VC2548 |  | -1.75 | 0.00000 | Bacterial surface antigen |
| VC2547 |  | -1.73 | 0.00000 | Hypothetical protein |
| VC0246 | *rfbH* | -1.72 | 0.00055 | Lipopolysaccharide O-antigen transport protein |
| VCA0124 | *tsiV3* | -1.72 | 0.00144 | T6SS |
| VCA0678 | *napA* | -1.65 | 0.00267 | Periplasmic nitrate reductase |
| VC1207 |  | -1.63 | 0.00000 | Hypothetical protein |
| VC0259 | *rfbV* | -1.62 | 0.00597 | Lipopolysaccharide biosynthesis protein |
| VC0633 | *ompU* | -1.62 | 0.00000 | Outer membrane protein |
| VC2516 |  | -1.62 | 0.00168 | Anti-sigma factor B antagonist |
| VC2517 |  | -1.62 | 0.00000 | Toluene tolerance |
| VC0287 | *gntV* | -1.60 | 0.01846 | Thermoresistance gluconokinase |
| VC1663 | *hslJ* | -1.60 | 0.00000 | Heat shock protein |
| VC1837 | *tolA* | -1.60 | 0.00000 | Membrane protein in colicin uptake |
| VCA0110 | *vasA* | -1.59 | 0.00002 | T6SS |
| VCA0679 | *napB* | -1.58 | 0.00849 | Nitrate reductase cytochrome c-type subunit |
| VC1763 |  | -1.57 | 0.00019 | Chemotaxis protein MotB-related |
| VC0090 | *dinF* | -1.56 | 0.00000 | DNA-damage inducible protein F |
| VC2221 |  | -1.56 | 0.00048 | Hypothetical protein |
| VCA0003 |  | -1.56 | 0.00135 | Hypothetical protein |
| VC0948 |  | -1.55 | 0.00000 | Rare lipoprotein A |
| VCA0928 |  | -1.55 | 0.02741 | Hypothetical protein |
| VCA0821 |  | -1.54 | 0.00000 | Hypothetical protein |
| VC1919 | *hupB* | -1.52 | 0.00018 | Histone-like DNA binding protein |
| VC2401 | *murG* | -1.52 | 0.00465 | Glycosyl transferase |
| VC1270 |  | -1.51 | 0.00000 | Glyoxylase II family protein |
| VCA0123 | *vgrG-3* | -1.50 | 0.00000 | T6SS |

^a^ORF IDs are derived from the *V. cholerae* N16961 genome.

^b^Fold change is the Δ*vxrB* mutant relative to the wild-type strain.
