# Supplementary material for: Vibrio cholerae Response Regulator VxrB Controls Colonization and Regulates the Type VI Secretion System
Source: PLoS Pathog. 2015 May 22;11(5):e1004933. doi: 10.1371/journal.ppat.1004933 (PMC4441509; doi:10.1371/journal.ppat.1004933)
Supplement: S3 Table — (DOCX) [file ppat.1004933.s008.docx]

**S3 Table. Genes negatively regulated by VxrB under AKI conditions**

| ORF ID^a^ | Gene | Fold Up Regulation^b^ | p-value | Predicted function |
| --- | --- | --- | --- | --- |
| VC1854 | *ompT* | 3.18 | 0.00000 | Outer membrane porin |
| VC2215 |  | 2.52 | 0.00000 | Cation transport |
| VC0713 |  | 2.39 | 0.01046 | Hypothetical protein |
| VC2216 | *copG* | 2.36 | 0.00000 | Copper-binding protein |
| VC0024 |  | 2.31 | 0.00282 | Sulphurtransferase |
| VC2561 | *cysG* | 2.31 | 0.01393 | Uroporphyrin-III C-methyltransferase |
| VC1740 |  | 2.22 | 0.00000 | Oxidoreductase, acyl-CoA dehydrogenase |
| VC1344 | *hppD* | 2.20 | 0.00000 | 4-hydroxyphenylpyruvate dioxygenase |
| VC1368 |  | 2.08 | 0.00005 | Hypothetical protein |
| VCA0913 | *hutB* | 2.08 | 0.01042 | Hemin ABC transporter |
| VC2559 | *cysN* | 2.01 | 0.00002 | Sulfate adenylate transferase |
| VCA0766 |  | 2.01 | 0.00063 | Cytochrome c554 |
| VC1332 |  | 2.00 | 0.00000 | Hypothetical protein |
| VC1737 | *infA* | 1.98 | 0.01954 | Initiation factor IF-1 |
| VC1823 | *frwB* | 1.96 | 0.00979 | PTS system, fructose-specific IIB |
| VC2231 | *fadE* | 1.95 | 0.00000 | Oxidoreductase, acyl-CoA dehydrogenase |
| VCA0260 |  | 1.94 | 0.00000 | Cupredoxin |
| VC1078 |  | 1.91 | 0.00602 | Hypothetical protein |
| VC0784 |  | 1.90 | 0.00000 | Sodium/alanine symporter |
| VCA0350 | *blc-2* | 1.90 | 0.00303 | Lipoprotein Blc |
| VCA0812 |  | 1.89 | 0.00000 | Leucine aminopeptidase-related protein |
| VC1345 |  | 1.85 | 0.00000 | Oxidoreductase |
| VC1927 | *dctM* | 1.83 | 0.00000 | C4-dicarboxylate transport protein |
| VC1565 |  | 1.80 | 0.00136 | Outer membrane efflux protein |
| VC1542 | *ligA-2* | 1.78 | 0.01349 | DNA ligase |
| VC2618 | *argD* | 1.78 | 0.00000 | Acetylornithine aminotransferase |
| VC1334 |  | 1.77 | 0.00003 | Hypothetical protein |
| VC2489 |  | 1.76 | 0.01042 | TetR family transcriptional regulator |
| VC1333 |  | 1.74 | 0.00128 | Hypothetical protein |
| VC1741 |  | 1.74 | 0.00390 | TetR family transcriptional regulator |
| VC2558 | *cysC* | 1.74 | 0.01196 | Adenylylsulfate kinase |
| VCA0293 |  | 1.74 | 0.00886 | Hypothetical protein |
| VC2617 |  | 1.72 | 0.00000 | Arginine/Ornithine succinyltransferase |
| VCA0567 | *vxrC* | 1.72 | 0.00000 | Hypothetical protein |
| VC2705 |  | 1.71 | 0.00000 | Sodium/solute symporter |
| VC1264 |  | 1.70 | 0.00002 | Iron-regulated protein A |
| VC0282 |  | 1.68 | 0.00000 | Methyl-accepting chemotaxis protein |
| VC1528 |  | 1.68 | 0.00565 | Periplasmic binding protein-like |
| VC1318 | *ompV* | 1.67 | 0.00002 | Outer membrane protein V |
| VCA0289 | *rpmI* | 1.67 | 0.00047 | Ribosomal protein L35 |
| VCA0616 | *folE* | 1.63 | 0.00057 | GTP cyclohydrolase |
| VC0069 |  | 1.62 | 0.00000 | Multidrug resistance protein |
| VC1928 |  | 1.62 | 0.00014 | C4-dicarboxylate transport protein |
| VC0787 |  | 1.61 | 0.02576 | LysR family transcriptional regulator |
| VC2177 |  | 1.61 | 0.04007 | Invasion gene expression up-regulator sirB |
| VC0706 |  | 1.60 | 0.00239 | Sigma-54 modulation protein |
| VC2704 |  | 1.60 | 0.00000 | Sodium symporter |
| VCA0925 | *pyrC* | 1.60 | 0.00001 | Dihydroorotase |
| VC0888 |  | 1.58 | 0.04712 | Pseudouridine synthase |
| VC1293 | *aspC* | 1.58 | 0.00004 | Aromatic amino acid aminotransferase |
| VCA0262 |  | 1.58 | 0.00568 | Hypothetical protein |
| VCA0749 | *glpC* | 1.58 | 0.00012 | Anaerobic glycerol-3-phosphate dehydrogenase |
| VC1506 |  | 1.57 | 0.00011 | Thioesterase |
| VCA0828 | *phhA* | 1.57 | 0.00146 | Phenylalanine-4-hydroxylase |
| VC0302 |  | 1.56 | 0.00371 | Transporter |
| VC1092 | *oppB* | 1.56 | 0.00000 | Oligopeptide ABC transporter |
| VC1547 |  | 1.53 | 0.01583 | Biopolymer transport protein |
| VC2758 | *fadB* | 1.53 | 0.00000 | Fatty oxidation complex |
| VC0018 | *ibpA* | 1.52 | 0.00001 | Heat shock protein A |
| VC1638 |  | 1.52 | 0.02834 | OmpR family response regulator |
| VCA0425 |  | 1.52 | 0.00256 | Hypothetical protein |
| VCA0889 |  | 1.52 | 0.00639 | LysR family transcriptional regulator |
| VC0263 |  | 1.51 | 0.00205 | Galactosyl-transferase |
| VC1897 |  | 1.51 | 0.00044 | Hisitidine triad protein |
| VC2341 |  | 1.51 | 0.00000 | Long chain fatty acid CoA ligase |
| VCA0767 |  | 1.51 | 0.00018 | TetR family transcriptional regulator |
| VCA1002 |  | 1.51 | 0.00884 | AzlC family protein |
| VC0425 |  | 1.50 | 0.00776 | Galactose-binding domain-like |
| VC2669 |  | 1.50 | 0.03708 | 5-carboxymethyl-2-2hydroxymuconate delta isomerase |

^a^ORF IDs are derived from the *V. cholerae* N16961 genome.

^b^Fold change is the Δ*vxrB* mutant relative to the wild-type strain.
